# Preclinical Evaluation of Bispecific Adaptor Molecule Controlled Folate Receptor CAR-T Cell Therapy With Special Focus on Pediatric Malignancies

**DOI:** 10.3389/fonc.2019.00151

**Published:** 2019-03-19

**Authors:** Yingjuan J. Lu, Haiyan Chu, Leroy W. Wheeler, Melissa Nelson, Elaine Westrick, James F. Matthaei, Ian I. Cardle, Adam Johnson, Joshua Gustafson, Nikki Parker, Marilynn Vetzel, Le-Cun Xu, Emilia Z. Wang, Michael C. Jensen, Patrick J. Klein, Philip S. Low, Christopher P. Leamon

**Affiliations:** ^1^Endocyte, Inc., West Lafayette, IN, United States; ^2^Ben Towne Center for Childhood Cancer Research (BTCCCR), Seattle Children's Research Institute, Seattle, WA, United States; ^3^Department of Chemistry, Purdue University, West Lafayette, IN, United States

**Keywords:** folate receptor, chimeric antigen receptor, CAR-T adaptor molecule, acute myeloid leukemia, osteosarcoma

## Abstract

Chimeric antigen receptor (CAR)-T cell therapy has transformed pediatric oncology by producing high remission rates and potent effects in CD19+ B-cell malignancies. This scenario is ideal as CD19 expression is homogeneous and human blood provides a favorable environment for CAR-T cells to thrive and destroy cancer cells (along with normal B cells). Yet, CAR-T cell therapies for solid tumors remain challenged by fewer tumor targets and poor CAR-T cell performances in a hostile tumor microenvironment. For acute myeloid leukemia and childhood solid tumors such as osteosarcoma, the primary treatment is systemic chemotherapy that often falls short of expectation especially for relapsed and refractory conditions. We aim to develop a CAR-T adaptor molecule (CAM)-based therapy that uses a bispecific small-molecule ligand EC17, fluorescein isothiocyanate (FITC) conjugated with folic acid, to redirect FITC-specific CAR-T cells against folate receptor (FR)-positive tumors. As previously confirmed in rodents as well as in human clinical studies, EC17 penetrates solid tumors within minutes and is retained due to high affinity for the FR, whereas unbound EC17 rapidly clears from the blood and from receptor-negative tissues. When combined with a rationally designed CAR construct, EC17 CAM was shown to trigger CAR-modified T cell activation and cytolytic activity with a low FR threshold against tumor targets. However, maximal cytolytic potential correlated with (i) functional FR levels (in a semi-log fashion), (ii) the amount of effector cells present, and (iii) tumors' natural sensitivity to T cell mediated killing. In tumor-bearing mice, administration of EC17 CAM was the key to drive CAR-T cell activation, proliferation, and persistence against FR+ pediatric hematologic and solid tumors. In our modeling systems, cytokine release syndrome (CRS) was induced under specific conditions, but the risk of severe CRS could be easily mitigated or prevented by applying intermittent dosing and/or dose-titration strategies for the EC17 CAM. Our approach offers the flexibility of antigen control, prevents T cell exhaustion, and provides additional safety mechanisms including rapid reversal of severe CRS with intravenous sodium fluorescein. In this paper, we summarize the translational aspects of our technology in support of clinical development.

## Introduction

Personalized medicine in pediatric oncology has reached a pivotal milestone with the recent approval of CD19-directed chimeric antigen receptor (CAR)-T cell therapies for certain B-cell malignancies (1, 2). The high remission rate (~70–90%) seen with this technology is not without serious side effects, including B-cell aplasia and high risk of cytokine release syndrome (CRS) (3). In addition, antigen-escape variants of the disease also contribute to relapses in some patients who require a different CAR-T cell product (4). On the other hand, continuing efforts are being made to develop similar modalities for acute myeloid leukemia (AML) and for pediatric solid tumors such as osteosarcoma (5, 6). Although AML is usually found in blood and bone marrow, it can seed skin and viscera and present as tumor masses termed chloromas (7, 8). Among early-phase clinical trials ongoing in adult AML patients, a CD123-directed CAR-T cell treatment (ClinicalTrials.gov identifier: NCT02159495) is showing promising antitumor activity with low grade CRS and no on-target/off-tumor toxicity reported to date (9). Since CD123 is a rare target overexpressed by AML blasts, a compound CAR T-cell strategy targeting both CD123 and CD33 is also being considered (10). However, CD33 is a normal myeloid surface antigen that likely requires gene editing to avoid myeloablative toxicity and severe CRS driven by on-target/off-tumor action of CD33 CAR-T cells (11). For osteosarcoma, suitable tumor targets are limited, but HER2, GD2/GD3, and NKG2D CAR-T cell treatments have been under investigation (5, 6, 12). For CAR-T cell therapies in general, solid tumors remain a significant therapeutic challenge due to the lack of ideal antigen targets, inter/intra-patient tumor heterogeneity, poor CAR-T cell infiltration/function, and the presence of an immunosuppressive microenvironment (13, 14).

The human folate receptor (FR) exists in multiple isoforms, but only FRα and FRβ are cell-surface glycoproteins with potential utility for cancer treatment. FRα is mostly expressed on epithelial cancer types including ovarian, cervical, renal cell carcinoma, non-small cell lung cancer, and triple-negative breast cancer (15). FRα is also present on the apical surfaces of a few normal epithelia (e.g., proximal tubules, choroid plexus, and bronchioalveolar cells) (16). FRβ is expressed on a few hematologic malignancies including AML and on a subset of tumor-associated macrophages (TAM) (17–19). An early report suggested that osteosarcoma frequently express FRα (20), and, FRβ + TAMs (internal communications) have also been observed in treatment-refractory osteosarcoma metastasis (21). Moreover, CAR-T cell therapies directed against FRα or FRβ have been explored using different antibodies and CAR constructs in human tumor xenograft models of ovarian cancer (22, 23), triple-negative breast cancer (TNBC) (24), gastric cancer (25), and AML (17). An early Phase 1 study with a FRα-specific CAR T cells (MOv19-4-1BB-CD3ζ) was conducted in ovarian cancer patients (26) and is being re-launched by intraperitoneal delivery (ClinicalTrials.gov identifier: NCT03585764).

**Figure 1:**
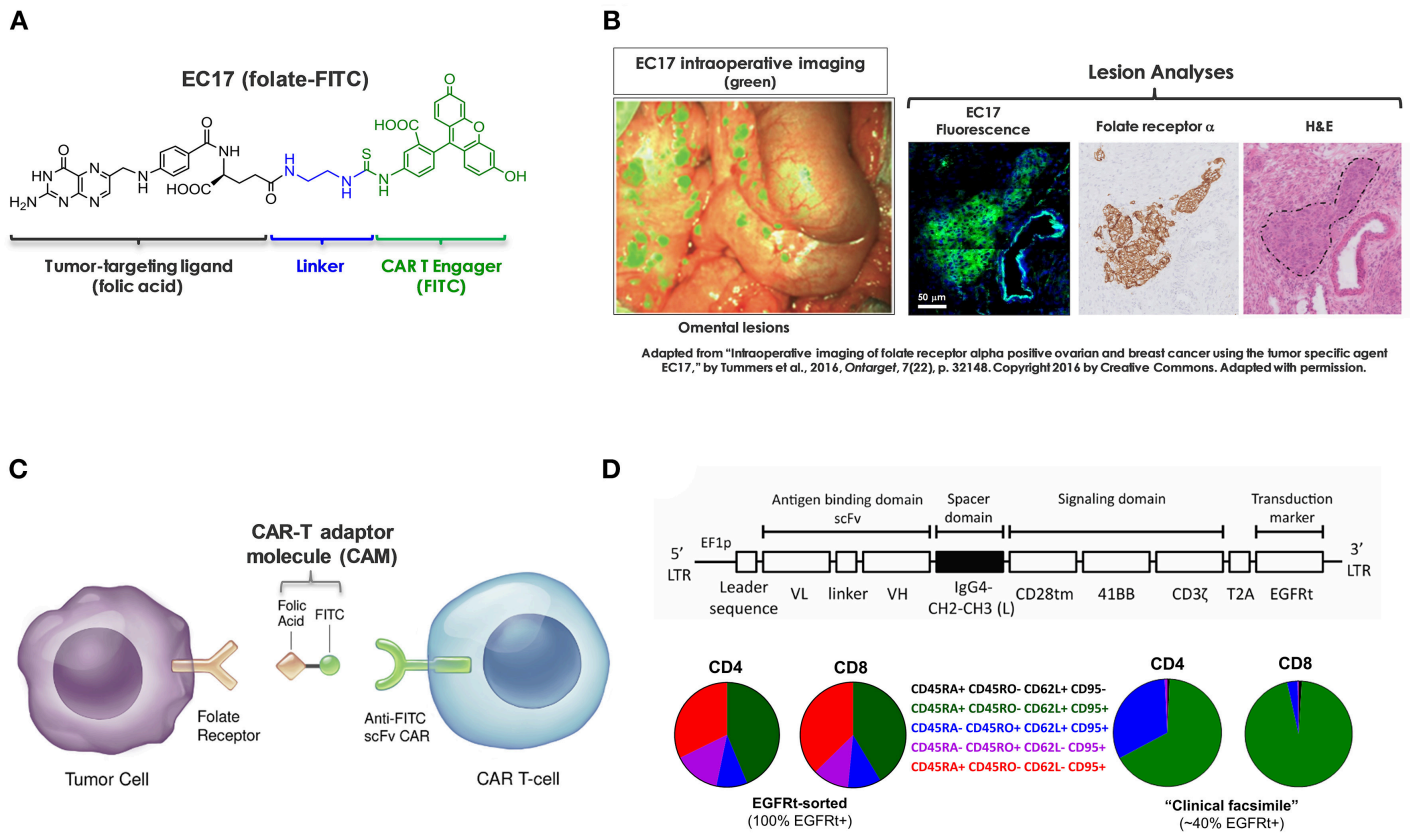
Components of CAR-T adaptor molecule (CAM)-based immunotherapy. **(A)** Chemical structure of EC17 CAM (folate-FITC, m.w. 873). **(B)** Ovarian cancer metastatic omental lesions identified by EC17 (green) during a fluorescence-guided surgery and subsequently confirmed of FRα expression by immunohistochemistry. **(C)** Diagram to show bispecific engagement of CAR-modified T cells with FR+ tumor cells. **(D)** Top: A fully human CAR construct comprised of anti-FITC scFv (clone E2), a full-length IgG4 spacer [Fc derived hinge-CH2(L235D, N297Q)-CH3], CD28tm transmembrane domain, 4-1BB/CD3ζ cytoplasmic activation domains, and a non-functional truncated cell surface polypeptide of epidermal growth factor receptor (EGFRt). Bottom: Examples of CD4/CD8 T cell phenotyping performed by flow cytometry on an EGFRt-sorted (left pie charts) CAR-T cell preparation and an unsorted “clinical facsimile” (right pie charts). The color keys are as shown.

Based on original research from Philip Low's laboratory at Purdue University, we are developing a CAR-T adaptor molecule (CAM)-based therapy that exploits a tumor-targeting ligand linked to a chemical CAR target antigen (fluorescein isocyanate, FITC) to redirect FITC-specific CAR-T cells against FR+ tumors (27). EC17 (folate-FITC, Figure 1A) is a low-molecular-weight ligand (m.w. 873) that has been given to renal cell carcinoma patients vaccinated against FITC (28, 29). In mice as well as in human subjects, EC17 penetrates solid tumors within minutes and is retained due to high affinity for the FR; whereas, unbound EC17 rapidly clears from the blood and from receptor-negative tissues (28, 30). Furthermore, EC17 was evaluated as an intraoperative imaging agent for real-time fluorescence-guided surgery sparing adjacent normal tissues (31, 32). In metastatic ovarian cancer patients, intravenously administered EC17 exquisitely “painted” the metastatic lesions embedded in omentum which were histologically confirmed to be FR+ malignant cells (Figure 1B). While employed as a bispecific CAM, EC17 serves the important function of “painting” FR+ tumors, engaging, and redirecting FITC-specific CAR-T cells in close proximity (Figure 1C). In NSG mice bearing human TNBC xenografts, EC17 plus CAR-T cell therapy showed curable antitumor activity. Subsequently, we found this approach was able to elicit FR-dependent antitumor activity with low or no adverse reactions against multiple cancer types of different histology, including ovarian cancer and renal cell carcinoma. But under defined conditions such as a high CAR-T cell dose and EC17 overtreatment, severe CRS could be induced (27). Further testing revealed that severe CRS can be mitigated or even prevented by gradually increasing subsequent EC17 doses following the initial CAR-T cell administration. Additionally, we also discovered that intravenous sodium fluorescein can be used to temporarily displace CAR-T cells from their targets to quickly reverse the CRS symptoms within hours (33).

Previously, we used a GFP+ second-generation anti-FITC scFv (clone 4M5.3) CAR containing the hinge and transmembrane sequences of CD8α and 4-1BB/CD3ζ signaling domains (i.e., FITC-4M5.3-scFv-CD8αhinge-CD8αtm-4-1BB/CD3ζ) (27, 33). For translation into first-in-human testing, a second-generation fully human FITC-specific (clone E2) CAR construct (herein refer to as E2-CAR in short) was synthesized (Figure 1D, top diagram) and CAR-modified T cells (Figure 1D, bottom pie charts) were provided by The Jensen laboratory at Seattle Children's Research Institute (SCRI) for preclinical evaluations (34). To establish proof-of-concept, we conducted a series of *in vitro* and *in vivo* studies using a range of FR+ and FR-negative tumor cell lines with special focus on those derived from pediatric AML and osteosarcoma. Using clinically relevant EC17 dosing regimens, we investigated key variables that contribute to the overall efficacy and risk of CRS toxicity in FR+ tumor models of TNBC, AML and osteosarcoma. As reported herein, these included CAR-T cell dose, EC17 dose/dose frequency, impact of dietary folate, tumor vs. tumor-free host, as well as pharmacokinetics and tumor uptake of CAR-T cells.

## Materials and Methods

### Cell Lines and Reagents

Unless otherwise noted, all FR+ and FR-negative cancer cell lines were, respectively, maintained in RPMI-1640 medium (Gibco BRL) supplemented with 10% heat-inactivated fetal calf serum without (FFRPMI) or with (RPMI) 2.4 μM folic acid (FA). KB (FRα-expressing human cervical carcinoma with HeLa markers) and CHO-β (Chinese hamster ovary cells transfected with human FRβ) were used as the sources of FRα and FRβ for radioligand binding assays, respectively (18). MDA-MB-231 represents a FRα+ subclone of human TNBC cell line. For AML studies, the green fluorescent protein (GFP)-expressing isogenic pairs of FRβ-positive (THP1-FRβ) and FR-negative (THP1-FG12) cell lines were kindly provided by Dr. Manohar Ratnam (The University of Toledo, Toledo, OH). Both were established from THP-1 (ATCC, TIB-202), a commonly used cell model for researching pediatric AML which was originally derived from a 1 year-old male infant with acute monocytic leukemia. For osteosarcoma studies, HOS-FRα was established by lentiviral transduction of FR-negative HOS-143b (ATCC, CRL8303) with FOLR1 gene encoding the human FRα. HOS-143b is originally established from a primary tumor of a 13 year-old Caucasian female and highly tumorigenic in NSG mice (35). The GFP-expressing bioluminescent pairs of FR+ HOS-FRα^fLuc^ and FR-negative HOS-143b^fLuc^ were transduced with lentiviral firefly luciferase and produced in the Jensen laboratory.

LEGENDplex™ human cytokine panels were purchased from BioLegend (San Diego, CA). The lactate dehydrogenase (LDH) based CytoTox 96® non-radioactive cytotoxicity assay kit was purchased from Promega (Madison, WI). Commercially available anti-human antibodies used for multicolor flow cytometry were: CD45RA (clone HI100), CD45RO (clone UCHL1), CD4 (clone SK3), and CD69 (clone FN50) from Thermo Fisher Scientific (Waltham, MA); CD3ε (clone SK7), CD8α (clone RPA-T8), CD137/4-1BB (clone 4B4-1), CD25 (clone M-A251), PD1 (clone EH12.1), LAG3 (clone T47-530), and TIM3 (clone 7D3) from BD Bioscience (San Jose, CA); biotinylated anti-human EGFR (Cetuximab, clone Hu1) from R&D systems (Minneapolis, MN); and FRα (clone LK26) from BioLegend (San Diego, CA). A fluorophore-conjugated anti-biotin was also purchased from BioLegend. APC-conjugated anti-FITC mouse IgG2a/kappa antibody (clone NAWESLEE), CountBright™ beads (Invitrogen), Annexin V staining buffer, and AlexaFluor-647-conjugated Annexin V were purchased from Thermo Fisher Scientific. For enzymatic digestion of tumor tissues, collagenase IV, hyaluronidase and DNase I were all purchased from Sigma-Aldrich (St. Louis, MO).

EC17 or folate-FITC [FA-(γ)-ethylenediamine-FITC] was synthesized at Endocyte. ^3^H-EC17 was either purchased from Moravek Biochemical (Brea, CA) at a specific activity of ~0.952 Ci/mmol or prepared at Endocyte by conjugating FITC with ^3^H-FA-(γ)-ethylenediamine made by ViTrax (Placentia, CA) at a specific activity of ~1.2 Ci/mmol. ^3^H-FA was also purchased from ViTrax at a specific activity of 59 Ci/mmol. For CRS rescue, sodium fluorescein dosing solution was diluted from AK-FLUOR® 25% (fluorescein injection, USP) which was purchased from Purdue Pharmacy (NDC 17478-250-25).

### Humanized CAR Construct and CAR-Modified T Cells

Developed by the Jensen team, a FITC-specific CAR construct was comprised of (1) a fully human anti-FITC scFv (clone E2, Kd = 0.75 nM), (2) an IgG4 hinge-CH2(L235D, N297Q)-CH3 spacer fused to a CD28-transmembrane domain (36), (3) a second-generation 4-1BB/CD3ζ-endodomain, and (4) a cell-surface human EGFRt tag, as published previously (37). To generate CAR-modified T cells, lentivirus was produced in 293T cells co-transfected with CAR-encoding epHIV7 lentiviral vector. Donor CD4+ and CD8+ T cells were purified by immunomagnetic selection and transduced separately or at a 50:50 ratio. In general, only one round of CD3/CD28 bead activation followed by one or two rounds of rapid *in vitro* expansion were carried out. For preclinical evaluations, several batches of EGFRt-sorted CD4, CD8 and unsorted CD4/CD8 CAR-T cells were used. All CAR-T cell preparations were analyzed prior to cryopreservation and after thawing to determine EGFRt expression and CD4/CD8 ratios by flow cytometry. Using combinations of surface markers, differentiation status of CD4+ and CD8+ CAR-T cell subsets on the day of infusion was analyzed and defined as T_N_, CD45RA+ CD45RO– CD62L+ CD95– naïve T cells; T_SCM_, CD45RA+ CD45RO– CD62L+ CD95+ stem cell memory T cells; T_CM_, CD45RA– CD45RO+ CD62L+ CD95+ central memory T cells; T_EM_, CD45RA– CD45RO+ CD62L– CD95+ effector memory cells; and T_EFF_, CD45RA+ CD45RO– CD62L– CD95+ effector T cells. For preclinical testing described below, we employed two batches of EGFRt-sorted pure CD4 and CD8 subsets (after mixing at 1:1 ratios) and two batches of unsorted ~1:1 EGFRt+ CD4/CD8 admixture including a “clinical facsimile” preparation with low differentiation profiles.

### EC17 CAM's Bispecific Affinity

The bispecific affinities of EC17 CAM were assessed using ^3^H-EC17 in cell-based radioligand binding assays. For binding to FR+ targets, KB and CHO-β cells were pre-seeded overnight in 24-well tissue culture plates and incubated with 0.1, 0.5, 1, 5, 10, 20, and 40 nM of ^3^H-EC17 in FFRPMI for 2 h at 37°C. Afterwards, the cells were rinsed with phosphate-buffered saline (PBS, pH 7.4) and lysed with 1% sodium dodecyl sulfate. The whole cell lysates were quantitated for the level of radioactivity and cellular protein content by standard Pierce BCA protein assay. The number of ^3^H-EC17 molecules bound per cell was calculated to determine the dissociation constants (Kd) for FRα (KB) and FRβ (CHO-β), respectively. To determine the Kd value of ^3^H-EC17 to effector cells, two million E2-CAR-T cells (100% EGFRt+, ~1:1 CD8/CD4 ratio) were incubated (1.5 h, room temperature) with 2-fold serial dilutions of ^3^H-EC17 (0–1,000 nM) with or without 500x excess nonradiolabeled “cold” EC17 in 150 μL of T cell culture medium [TexMACS™ medium (Miltenyi biotec, Auburn, CA) supplemented with 2% heat-inactivated human AB serum]. Afterwards, the cells were washed 4x with PBS, lysed with 0.1% Triton X-100 plus 0.1 N NaOH and counted in a scintillation counter. The specific binding curve was generated by subtracting non-specific binding (^3^H-EC17 plus “cold” EC17) from total binding (^3^H-EC17) at each concentration of the radioligand.

### Co-culture Experiments

To investigate EC17 CAM action *in vitro*, pure EGFRt-sorted CD4 and CD8 CAR-T cells were admixed in a 1:1 ratio and matching mock-transduced T cells were included per experimental design. Other than FR+/- isogenic pairs of AML and osteosarcoma cell lines, we added KB, MDA-MB-231 and OV90 to represent different histological tumor types and FR expression levels. All FR+ target cell lines were subjected to a direct ^3^H-FA binding assay to quantitate the number of FR molecules bound/cell (38). Prior to co-culture experiments, cryopreserved CAR-T cells were allowed to recover for 2–3 days in T cell culture medium. All effector and target cells were pre-washed with FFRPMI to remove any exogenous FA that could compete against EC17. Short-term co-culture studies of ≤ 3 days were carried out in FFRPMI without adding any exogenous cytokines.

For an EC17 dose response study, FR+ cancer cell lines (KB, MDA-MB-231, HOS-FRα, THP1-FRβ, OV90) were co-cultured with EGFRt-sorted E2-CAR-T cells at an effector-to-target (E/T) ratio of 1:1 in the presence of EC17 ranging from 0.1 pM to 100 μM in 10-fold increments. After 24 h of co-culture, supernatants were harvested to determine target cell killing by Promega's CytoTox 96® LDH assay kit and expressed as specific lysis (%) normalized to basal levels in the absence of EC17. To study the kinetics of T cell activation and FR correlation *in vitro*, co-cultures were carried out in FR+/- cell lines at varying E/T ratios (1:27, 1:9, 1:3, 1:1, 3:1) in the continuous presence of 10 nM EC17. The kinetics of target cell killing (i.e., % specific lysis) were quantified after 16 and 48 h of co-culture and normalized to basal levels in the absence of EC17 at defined E/T ratios. Meanwhile, a portion of the supernatants was taken after 44 h of co-culture for measurement of IFNγ, IL-2, and TNFα. The LEGENDplex™ human Th1 cytokine panel (BioLegend, San Diego, CA) was used to determine CAR-T cell derived cytokines.

To study CAR-T cell activation in the presence of FR+ tumor cell targets, co-culture experiments were initially carried out with MDA-MB-231, THP1-FRβ, and HOS-FRα. After a 30 min exposure to media containing 100 nM EC17, all target cells were washed and then incubated at 1:1 E/T ratio with EGFRt-sorted E2-CAR-T cells (1:1 CD4/CD8) or mock transduced control T cells. After 24 h of co-culture, the floating and adherent cells were harvested and pooled. The surface expression of T cell activation markers CD69, CD137 (4-1BB), and PD1 were analyzed by flow cytometry. For T cell exhaustion studies, FR+ (KB, MDA-MB-231, HOS-FRα^fLuc^, THP1-FRβ) and FR-negative (HOS-143b^fLuc^, THP1-FG12) tumor cell lines were used as targets. Here, target cells were incubated on day 0 without or with 0.1 and 10 nM of EC17 for 30 min at 37°C and the status of EC17 “pre-loading” was assessed by counterstaining cell-surface EC17 with an anti-FITC antibody. Three days later, the surface expression of PD1, LAG3, and TIM3 inhibitor receptors on co-cultured CAR-T cells were analyzed and higher frequencies of double- and triple-positive cells were considered approaching an exhausted phenotype (39). As tumor cells used in this study showed variability of doubling times, target cell apoptosis (%) was measured as Annexin V+ after 2 days of co-culture and normalized against background levels of apoptosis of target cells cultured under the same conditions in the absence of T cells.

### Tumor Models

All animal care and use were performed according to NIH guidelines and in compliance with protocols approved by the Purdue University Animal Use and Care Committee. Female 4 to 5-weeks-old NOD/SCID gamma (NSG™) mice (stock number: 005557) were purchased from The Jackson Laboratory (Bar Harbor, ME). Unless specifically indicated, all animals were maintained on a FA-deficient diet (TestDiet, St. Louis, MO) upon arrival and throughout the study. To establish subcutaneous xenografts, MDA-MB-231 and HOS-FRα were implanted in the right flank region at 2.5 × 10^6^ and 1 × 10^6^ cells per animal, respectively. For intravenous xenografts, THP1-FRβ cells were inoculated at 5 × 10^6^ cells per animal. Subcutaneous tumors were measured 2–3 times per week with a caliper and calculated using the ellipsoidal formula (length x width^2^)/2. Euthanasia was performed per study design or when (i) the animals had lost ≥20% of body weight or approached moribund conditions, (ii) subcutaneous tumors reached ≥1,500 mm^3^ in size, or (iii) animals displayed signs of swollen belly and severe distress (i.e., THP1-FRβ). All animal doses (CAR-T cells, EC17, sodium fluorescein) were given intravenously.

### Kinetics of CAR-T Cell Expansion and Tumor Uptake

To study CAR-T cell expansion *in vivo*, we started with 15 MDA-MB-231 tumor bearing mice having ~4.8 million EGFRt-sorted E2-CAR-T cells (1:1 CD4/CD8) injected on day 0. When the tumor sizes averaged at ~350 ± 60 mm^3^ on day 2, up to 6 weekly doses of EC17 at 500 nmol/kg were given on days 2, 9, 16, 23, and 30. For *ex-vivo* analysis, 3 animals were taken before the next EC17 dose on days 9, 16, 23, and 30 for a total of 4 collections. For comparison, we also gave the same EC17 dose to 3 CAR-T mice that had ~4.4 × larger tumor sizes on day 2 (~1555 ± 79 mm^3^). For each collection, fresh blood and tumor samples (if available) were analyzed for the presence of human CD3ε+ EGFRt+ CAR-T cells by flow cytometry. Moreover, we analyzed phenotypic changes of E2-CAR-T cells in the circulation (T_SCM_, T_CM_, T_EM_, and T_EFF_) and the activation status of tumor-infiltrating CAR-T cells (i.e., CD137/4-1BB, PD1).

### Flow Cytometry *ex-vivo*

For CAR-T cell analysis in the circulation, plasma was removed from a predetermined volume of whole blood collected into tubes containing ethylenediaminetetraacetic acid anticoagulant. After lysing red blood cells, leukocyte pellets were re-suspended in a flow cytometry staining solution comprising 1% bovine serum albumin, 50 mg/mL human IgG (Equitech Bio) and 0.09% sodium azide. The samples were stained for human leukocyte surface markers (CD3ε, CD4, CD8α, CD45RA, CD62L) and biotinylated anti-human EGFR (cetuximab) followed by a fluorophore-conjugated anti-biotin secondary antibody. For analysis of tumor-infiltrating CAR-T cells, pre-weighed fresh tumor fragments were finely minced and enzymatically digested with a digestion cocktail consisting of 0.5 mg/mL collagenase IV, 0.5 mg/mL hyaluronidase and 0.1 mg/mL DNase I in serum-free FFRPMI with vigorous shaking for 1 h at 37°C. Afterwards, tumor cell pellets underwent a red blood cell lysis step, washed with cold PBS and filtered through a 40 μm nylon cell strainer. The resulting single cell suspensions were stained for EGFRt and human leukocyte markers, CD137/4-1BB and PD1. A minimum of 20,000 propidium iodide negative live cell events were collected on the Gallios flow cytometer and analyzed with the Kaluza software, version 2.1 (Beckman Coulter, Brea, CA). CAR-positive T cells were identified in mouse blood as human CD3ε+ EGFRt+ events and absolute numbers per volume of blood were calculated using equal numbers of CountBright™ beads (Invitrogen, Carlsbad, CA) added to each sample. The number of tumor-infiltrating CD3ε+ EGFRt+ CAR-T cells was expressed as % total viable tumor cells analyzed.

### Tumor Therapies

In a therapeutic setting, EC17 CAM can theoretically be given before or after CAR-T cell injection (33). For all intended purposes here, we administered the first dose of EC17 2–3.5 days after CAR-T cells to allow for an observation period of human T cells in tumor-bearing mice. Two batches of unsorted E2-CAR-T cells (23% or 39% EGFRt+, 1:1 CD4/CD8) were used for *in vivo* studies. On the day of infusion for each experiment (day 0), frozen CAR-T cells were quickly thawed at 37°C, washed 2x with Dulbecco's 1X PBS (pH 7.4) and injected into the tail veil at desired EGFRt+ E2-CAR-T cell doses. In addition, a small aliquot of CAR-T cells was analyzed by flow cytometry for CD4 to CD8 ratio and differentiation status of CAR-T cells. On the first day of EC17 dose, tumor-bearing animals were randomly assigned into groups according to their tumor sizes or the same number of days post intravenous implantation (i.e., THP1-FRβ).

For MDA-MB-231 studies, mice received a high dose (~10 million) of a “clinical facsimile” of E2-CAR-T cells (~39% EGFRt+) with a low differentiation profile. Two days later, we started them with 3 different treatment regimens of EC17 at an average tumor size of ~293 ± 39 mm^3^. The EC17 dosing was given once-a-week (SIW) at 500 nmol/kg on Mondays, or as escalating doses of 5, 50, or 100, and 500 or 1,000 nmol/kg on Monday, Thursday, and Monday with a 6 day break in-between cycles. Control mice were left untreated (received CAR-T cells but no EC17). For comparison, a cohort of tumor-free littermates also received the same number of CAR-T cells without or with EC17 SIW at 500 nmol/kg. To study the effect of high dietary folate, two sets of NSG mice were placed on either a FA-replete diet (4 mg/kg, Envigo, Indianapolis, IN) or a FA-deficient diet (TestDiet, St. Louis, MO) upon arrival. On day 0, both sets of the mice received ~10 million of the same “clinical facsimile” CAR-T cells followed by no EC17 or EC17 SIW at 500 nmol/kg starting on day 3. On the first day of EC17 administration, MDA-MB-231 tumors size averaged around ~549 ± 184 mm^3^ (280–918 mm^3^) in FA-replete mice, and ~559 ± 165 mm^3^ (356–961 mm^3^) in FA-deficient mice.

For AML studies, mice were intravenously infused with THP1-FRβ tumor cells 1 day prior to receiving a low dose of ~6 million E2-CAR-T cells (~23% EGFRt+). At ~3.5 days post CAR-T cell infusion, EC17 was dosed in 3 different ways including (i) SIW at 500 nmol/kg, (ii) thrice at 5/50/500 nmol/kg on Monday/Wednesday/Friday followed by a 9 day break in between cycles (TIW On/Off), and iii) as escalating doses of 5/10/100 nmol/kg in Cycle 1, 5/30/300 nmol/kg in Cycle 2, and 5/50/500 nmol/kg in Cycle 3, all on Monday/Thursday/Monday with a 6 day break in between cycles (M/Th/M, On/Off). On day 31, satellite animals were harvested for quantification of CAR-positive T cells identified in the mouse blood as human CD3ε+ EGFRt+ events and calculated as absolute numbers per 100 μL of whole blood. Upon euthanasia or at the end of study on day 38, total tumor load in THP1-FRβ tumor-bearing mice was assessed by measuring GFP+ tumor cells in the blood by flow cytometry and collecting liver weight (with metastatic lesions) and total weight of nonliver macrometastases found in the body cavities. Tumor fragments of liver metastases were enzymatically digested into single-cell suspensions and viable cell populations were analyzed for the status of CAR-T cell exhaustion using anti-human PD1, LAG3, and TIM3 (clones EH12.1, T47-530, and 7D3, respectively).

For osteosarcoma study, two cohorts of mice were subcutaneously implanted with HOS-FRα tumor cells 3 days prior to receiving ~6 million of the same CAR-T cell preparation used in the THP1-FRβ study. At ~3.5 days post CAR-T cell infusion, one cohort of mice was given up to 3 cycles of EC17 at 5/10/100 nmol/kg in Cycle 1, 5/30/300 nmol/kg in Cycle 2, and 5/50/500 nmol/kg in Cycle 3, all following the Monday/Thursday/Monday regimen with a 6 day break in-between cycles. At the end of study, circulating CD3ε+ EGFRt+ CAR-T cells per 100 μL of mouse blood were enumerated. HOS-FRα tumors (+/- EC17 treatment) were also harvested and digested for flow cytometric analysis of tumor-infiltrating CAR-T cells.

### Toxicity and CRS Rescue

Depending on the CAR-T cell dose and how EC17 is administered, tumor-bearing mice receiving FITC-specific CAR-T cells can experience severe CRS and varying degrees of body weight loss (27, 33, 34). Therefore, we developed a CRS grading system (0–5 scale) to empirically assess CRS toxicity from animals' gross morphology and social behavior to allow for better timing of CRS rescue. While grades 0 and 5 indicated normal (no CRS) or death (due to severe CRS), respectively, grades 1, 2, and 3–4 were considered mild, light-to-moderate, and severe CRS. In addition, all EC17 doses were intentionally given toward the end of any given day to allow potential CRS symptoms to develop overnight. On the days immediately following each EC17 dose, animals were scored and rescue agents such as sodium fluorescein, folic acid and leucovorin were used to mitigate CRS toxicity (33). In this particular instance, a batch of E2-CAR-T cells with a high EGFRt+ CD4/CD8 ratio (~93:7) was given to HOS-FRα tumor-bearing mice at a mixture of ~2.5 million CD8 and ~33 million CD4 on day 0. When EC17 was dosed, respectively, at 500, 100, and 500 nmol/kg on days 3, 10, and 12, low CRS (grades 1–2) was noted on days 4 and 11 but high CRS (grade 3) was noted on day 13. To mitigate the severe CRS toxicity, sodium fluorescein was intravenously administrated at ~96 mg/kg in the morning of day 13 and mouse plasma samples were collected 6 h later for analysis of CRS-associated cytokines.

### Statistics

Statistical analyses were performed using the computer program GraphPad Prism (GraphPad Software Inc., San Diego, CA). Data were analyzed using Student's *t*-test or one-way ANOVA. If applicable, data were further analyzed across treatment groups using appropriate multiple comparison post-test. ^*^p < 0.05 was considered statistically significant in all tests.

## Results

### Principal Components of CAM-Controlled CAR-T Cell Therapy

**Figure 2:**
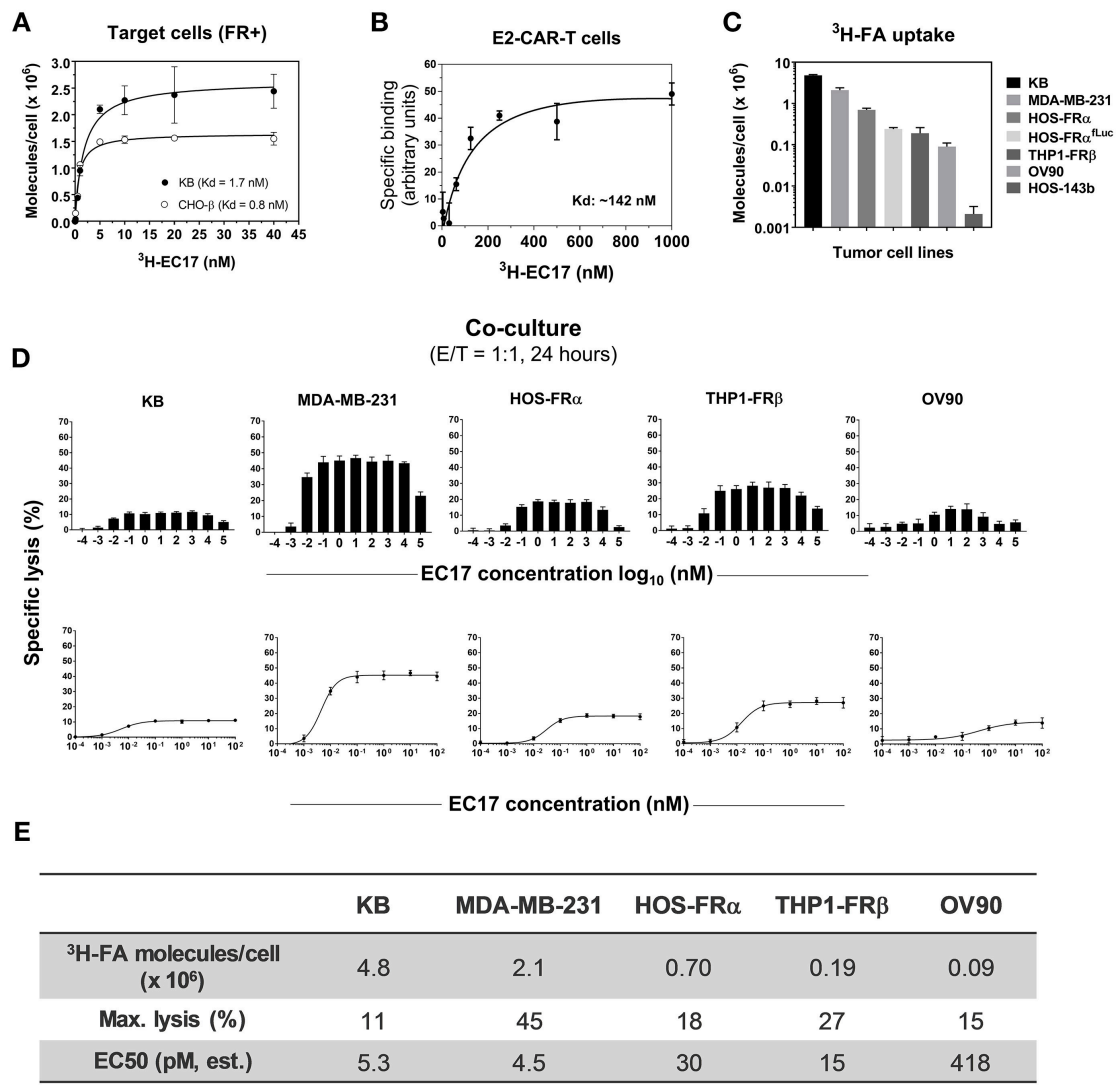
EC17 CAM's bispecific affinity and dose response in CAR-T cell activity against FR+ tumor targets *in vitro*. **(A)** Kd values of ^3^H-EC17 uptake by FR+ target cells after a 2 h incubation at 37°C (calculated from the numbers of molecules bound per cell). **(B)** Kd value of ^3^H^−^EC17 uptake by E2-CAR-T cells (~100% EGFRt+, 1:1 CD8/CD4 ratio) after a 1.5 h incubation at room temperate (estimated from the specific binding curve). **(C)** Shown are functional FR levels on tumor cell measured by a ^3^H-FA-based binding assay (100 nM, 1 h at 37°C). **(D)** Full-range EC17 dose response in specific lysis (%) of 5 FR+ tumor cell lines co-cultured with EGFRt-sorted CAR-T cells for 24 h at 1:1 E/T ratio. **(E)** Maximum lysis (%) and EC50 values were obtained from the dose-response curves fitted up to 100 nM.

The basic components of our CAR-T cell therapy involve FITC as the pseudo tumor antigen, the high affinity EC17 CAM, and a rationally designed anti-FITC CAR and CAR-modified T cells (Figure 1). To our advantage, the bispecific engager, EC17, has already been tested in the clinic for immunotherapy and optical imaging purposes (28, 29, 32, 40). To directly quantify its bispecific binding affinities, ^3^H-EC17 was synthesized and radioligand binding assays were carried out on KB and CHO-β cell lines representing FRα+ and FRβ+ target cells, respectively, and on E2-CAR-T cells representing the effector cells. When binding to its targets, EC17 demonstrated similar affinities toward both FRα and FRβ with low Kd values of 1.7 and 0.8 nM, respectively (Figure 2A). Upon binding to unsorted E2-CAR-T cells (~100% EGFRt+, 1:1 CD8/CD4 ratio), the Kd value was estimated at ~142 nM (Figure 2B).

Amid a series of different CAR constructs synthesized and evaluated by the Jensen team, a fully human anti-FITC scFv (FITC-E2) CAR was chosen for preclinical development (Figure 1D). This second-generation fully human CAR consisted of anti-FITC scFv (clone E2), an IgG4-Fc spacer/hinge with double mutations in the CH2 region (L235D and N297Q) to reduce binding to FcγR (36), a CD28 transmembrane domain, and 4-1BB/CD3ζ signaling domains appended to a cell-surface EGFRt tag by a T2A ribosomal skip sequence (i.e., FITC-E2-scFv-IgG4hinge-CD28tm-4-1BB/CD3ζ-T2A-EGFRt). For preclinical studies, both EGFRt-sorted and unsorted E2-CAR-T cells were prepared at ~1:1 CD4/CD8 ratios, and T cell subtype phenotyping was routinely performed by flow cytometry at the time of CAR T cell infusion (day 0) for each *in vivo* experiment. A typical expression pattern of EGFRt-sorted CAR-T cells was comprised of both CD4 and CD8 subsets at ~42% T_SCM_, 10% T_CM_, 12% T_EM_, and 34% T_EFF_ (Figure 1D, pie charts on the left). Only EGFRt-sorted CAR-T cells were used for co-culture and pharmacokinetic studies. For tumor therapy, we used a “clinical facsimile” batch with a low differentiation profile (Figure 1D, pie charts on the right) for MDA-MB-231 studies and a research batch for THP1-FRβ and HOS-FRα studies. The “clinical facsimile” batch (~39% EGFRt+) was comprised of CD4+ subsets at ~66% T_SCM_ and ~32% T_CM_ and CD8 subsets at ~95% T_SCM_ and 3% T_CM_. The research batch (~23% EGFRt+) was more differentiated and comprised of CD4 subsets at 32% T_SCM_, 53% T_CM_, 11% T_EM_, and 3.7% T_EFF_ and CD8 subsets at 44% T_SCM_, 0.28% T_CM_, 3.4% T_EM_, and 52% T_EFF_.

### Functional FR Assessments

Besides pediatric cancer cell lines transfected with FRα (HOS-FRα) and FRβ (THP1-FRβ), we included cancer cell lines of different histology and FR expression levels (Figure 2C). As estimated by a radioligand binding assay (100 nM ^3^H-FA, 1 h at 37°C), the ranking order of total available FRs on these cell lines was: 9 × 10^4^ (OV90, a low-FR expressing ovarian cancer cell line), 1.9 × 10^5^ (THP1-FRβ), 2.4 × 10^5^ (HOS-FRα^fLuc^), 7 × 10^5^ (HOS-FRα), 2.1 × 10^6^ (MDA-MB-231), and 4.8 × 10^6^ (KB) FA molecules/cell. Also included as FR-negative controls were HOS-143b^(fLuc)^ and THP1-FG12 parent cell lines. Thus, the general ranking of functional FR expression on co-cultured FR+ cancer cell lines was: KB > MDA-MB-231 > HOS-FRα > HOS-FRα^fLuc^ > THP1-FRβ (AML) > OV90.

### High Potency of EC17 CAM in Effector/Target Cell Engagement *in vitro*

Monovalent EC17 in its free form (i.e., one FITC per FA ligand) does not automatically activate anti-FITC CAR-T cells (27). To study the robustness of CAR-T cell activation, 5 FR+ cell lines (KB, MDA-MB-231, HOS-FRα, THP1-FRβ, OV90) were used along with a wide range of EC17 concentration (0.1 pM to 100 μM) at an E/T ratio of 1:1 (Figures 2D,E). Specific lysis (%) was quantified by Promega's LDH cytotoxicity assay kit after 24 h of co-culture. In all cell lines, EC17-dependent cytolysis followed a bell-shaped dose response with a somewhat broad concentration plateau (~0.1 nM to 1 μM) (Figure 2D). When the dose-response curves were fitted to the summit (up to 100 nM), half-maximal effective concentrations (EC_50_) were obtained at 4.5 pM (MDA-MB-231), 5.3 pM (KB), 15 pM (THP1-FRβ), 30 pM (HOS-FRα), and 418 pM (OV90), respectively. However, maximal lysis obtained for each cell line ranked differently at 45% (MDA-MB-231), 27% (THP1-FRβ), 18% (HOS-FRα), 15% (OV90), and 11% (KB) (Figure 2E). The low EC50 values in general suggested that EC17 was highly potent in triggering FR-specific target cell lysis by activated E2-CAR-T cells.

### Correlation of FR Levels With Target Cell Lysis and Cytokine Production

**Figure 3:**
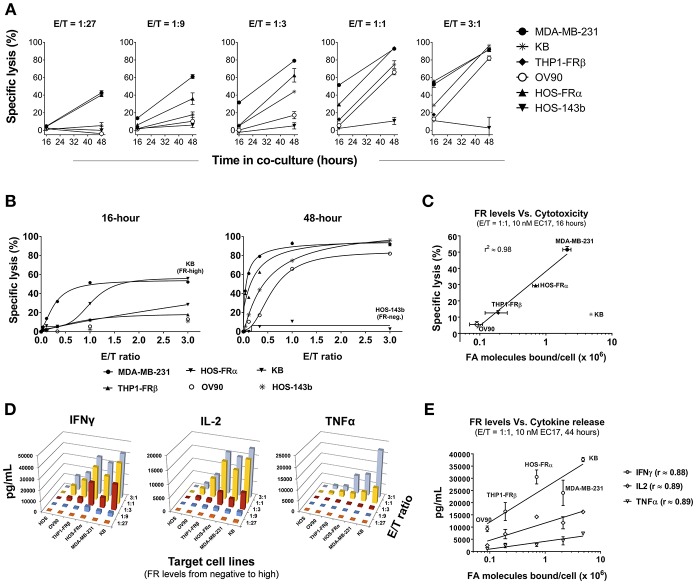
Correlation of CAR-T cell activity with FR levels and tumor cells' natural sensitivity. **(A)** Kinetics of specific lysis (%) at varying E/T ratios in FR+ (MDA-MB-231, KB, THP1-FRβ, OV90, HOS-FRα) and FR-negative (HOS-143b) cell lines after 16 and 48 h of co-culture in the presence of 10 nM EC17. **(B)** Specific lysis (%) of target cells plotted against a linear scale of E/T ratios. High FR+ KB cells demonstrated an early resistance at 16 h while FR-negative HOS-143b failed to respond. **(C)** Excluding KB cells, a semi-Log correlation was established between specific lysis (%) at 16 h of co-culture and functional FR levels on tumor cells. **(D)** 3D diagrams depicting the relationships between the levels of CAR-T cell derived Th1 cytokines (IFNγ, IL-2, and TNFα) after 44 h of co-culture at 10 nM EC17 with varying E/T ratios. **(E)** CAR-T cell derived Th1 cytokine levels plotted against FR levels of FR+ target cell in a Log10 scale.

To expand our investigation into FR dependency, 5 FR+ (MDA-MB-231, KB, HOS-FRα, THP1-FRβ, OV90) and 1 FR-negative (HOS-143b) cell lines were co-cultured in the continuous presence of 10 nM EC17 with E2-CAR-T cells at varying E/T ratios (1:27, 1:9, 1:3, 1:1, and 3:1). Specific lysis (%) was quantified after 16 and 48 h of co-culture, and Th1 cytokines (IFNγ, IL-2, TNFα) in culture media were measured after 44 h of co-culture. While the onset of specific lysis (~16 h) varied among different tumors, an increase in specific cytolysis was observed at 48 h for all FR+ cancer cell lines (Figure 3A). It generally took a longer time (48 h) and a higher E/T ratio (≥1:1) to see significant activity in the lowest FR-expressing OV90 cells (Figure 3A). Interestingly, the high FR-expressing KB cells responded very slowly and also required a higher E/T ratio (≥1:3) and longer exposure time to cause significant cell death (Figure 3B, left plot). When enough effector cells were present (≥1:1 E/T ratios), all FR+ cancer cells responded after 48 h of co-culture. Notably, only the FR-negative HOS-143b cells were essentially unharmed (Figure 3B, right plot).

To establish a correlation of CAR-T cell activity with FR expression *in vitro*, we plotted the values of 16 h specific lysis at an E/T ratio of 1:1 against the functional FR levels found on tumor cells (Figure 2C). Excluding KB cells that showed an unusual resistance at the beginning, there was a strong semi-log correlation between FR expression and the onset of cytolysis with an *R*-value of 0.98 (Figure 3C). Similarly, all but the FR-negative HOS-143b tumor cells triggered high levels of IFNγ, IL-2, and TNFα production after 44 h of co-culture at an E/T ratio of 1:1 with E2-CAR-T cells (Figure 3D). Interestingly for Th1 cytokine production, a semi-log correlation was also observed with all cell lines included at R-values of 0.88 (IFNγ), 0.89 (IL-2), and 0.89 (TNFα), respectively (Figure 3E). In short-term co-cultures, MDA-MB-231 was found most sensitive to killing by CAR-T cells, but HOS-FRα osteosarcoma and THP1-FRα AML also responded quickly in a FR level-dependent manner (Figure 3C). Together, these data suggested that (i) CAR-T cell is activated only when EC17 forms the intercellular bridge between CAR-T cell and tumor cell, (ii) other factors such as E/T ratio, exposure time and tumor sensitivity confer the overall activity of CAR-T cell against a particular FR+ tumor target, and (iii) too much EC17 would interfere with CAR-T cell engagement leading to decreased target cell death.

### EC17/FR-Dependent CAR-T Cell Activation and Exhaustion *in vitro*

**Figure 4:**
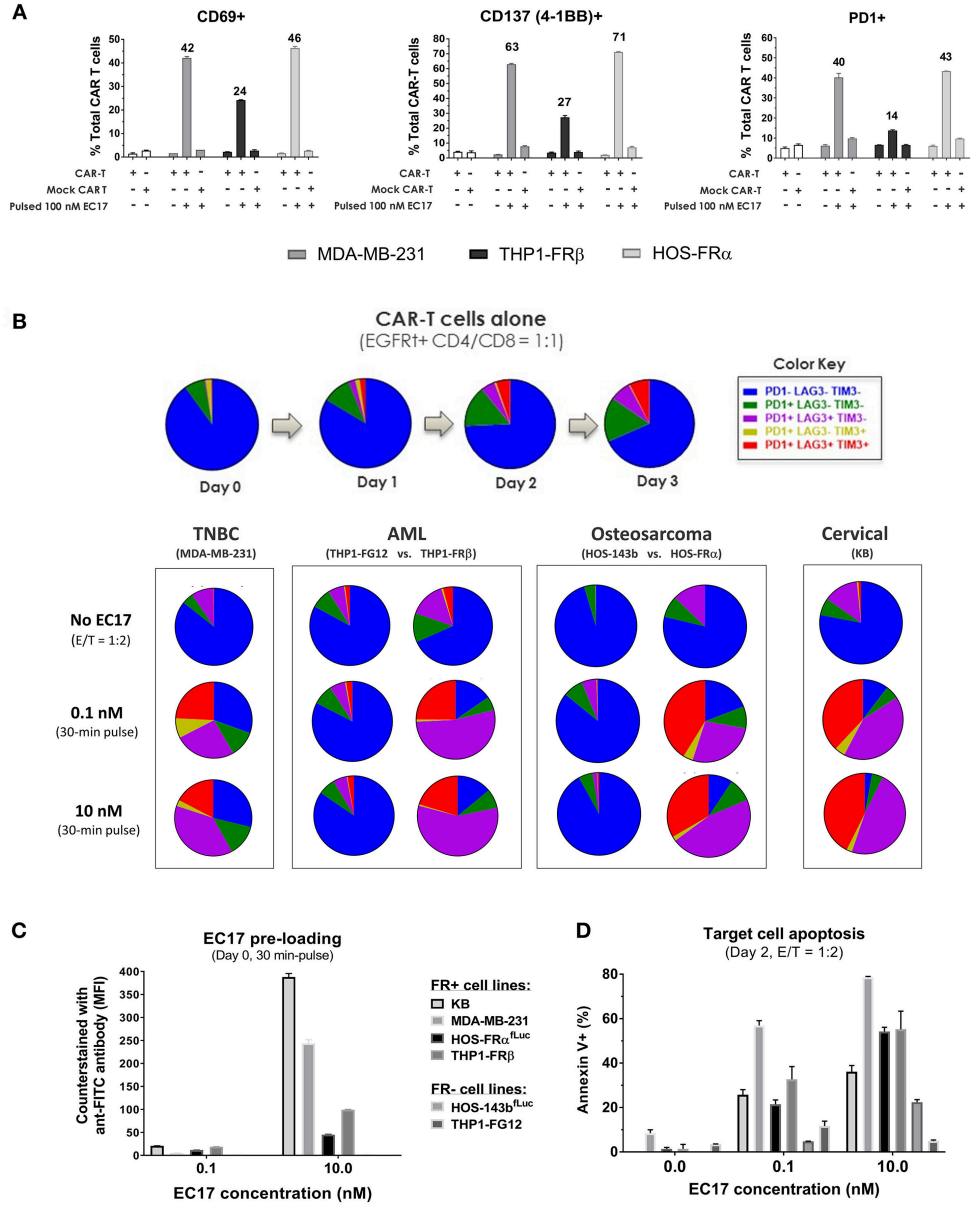
EC17/FR-dependent CAR-T cell activation/exhaustion in different tumor types. **(A)** Expression of early (CD69), intermediate (CD137), and late (PD1) T cell activation markers detected on CAR-T cells co-cultured (E/T = 1:1) with FR+ tumor cell targets pre-loaded without or with EC17 (100 nM, 30 min pulse at 37°C). The first two open bars represent CAR-T and Mock transduced T cells only without target cells. **(B)** Top row: Colored pie charts representing change in the differentiation status of CAR-T cells in culture without target cells on days 0–3. Bottom row: Four sets of colored pie charts representing the differentiation status of CAR-T cells after 3 days of co-culture (E/T = 1:2) with FR+ (MDA-MB-231, THP-1FRβ, HOS-FRα, KB) and FR-negative (THP1-FG12, HOS-143b) tumor cells preloaded without or with EC17 (0.1 or 10 nM, 30 min pulse at 37°C). **(C)** Bar graph to show EC17 loading status of target cells confirmed by flow cytometry and expressed as MFI (EC17 was undetectable on FR-negative cell lines). **(D)** Target cell apoptosis (%) detected by Annexin V staining after 2 days of co-culture as described in **(B)**.

To examine antigen-dependent CAR-T cell activation, we initially pre-loaded MDA-MB-231, THP1-FRβ, and HOS-FRα cells with 100 nM EC17 over 30 min. The cells were washed and then incubated at 1:1 E/T ratio with either EGFRt-sorted E2-CAR-T cells (1:1 CD4/CD8), or mock transduced control T cells. After 24 h of co-culture, we measured the surface expression of T cell activation markers CD69, CD137 (4-1BB), and PD1 (Figure 4A). EC17-preloaded MDA-MB-231 and HOS-FRα were strong activators of our CAR-T cells. THP1-FRβ cells, which express 11-fold lower FR compared to the MDA-MB-231 cells, did trigger CAR-T cell activation, but at lower levels. Importantly, the mock control T cells were not activated under these conditions. As some FR+ tumor types (e.g., KB) display a natural resistance to killing by EC17-directed CAR T cells (Figures 2, 3), we studied T cell activation vs. exhaustion as a mechanism of EC17 CAM action. Both FR+ (KB, MDA-MB-231, HOS-FRα^fLuc^, THP1-FRβ) and FR-negative (HOS-143b^fLuc^, THP1-FG12) tumor cell lines were chosen for this purpose. HOS-FRα^fLuc^ expressed ~5.6x lower level of functional FR than its parent HOS-FRα, and HOS-143b^fLuc^ was FR-negative similar to HOS-143b (Figure 2C). Here, FR+ and FR-negative tumor cells without or with EC17 pre-loading (0.1 or 10 nM, 30 min pulse at 37°C) were incubated with EGFRt-sorted CAR-T cells (1:1 CD4/CD8) at an E/T ratio of 1:2 (Figures 4B,D). EC17 dose-dependent pre-loading was confirmed on day 0 by counter-staining of membrane bound EC17 with an APC-conjugated anti-FITC antibody (clone NAWESLEE) (Figure 4C). Three days later, co-cultured CAR-T cells were measured for upregulation and co-expression of T cell activation/exhaustion markers PD1, LAG3, and TIM3. In the absence of target cells, E2-CAR-T cells underwent a low-grade differentiation in culture with low frequencies of double or triple-positive cells. Interestingly, the presence of tumor cells alone (without EC17 pre-loading) appeared to “relax” the CAR-T cells to various degrees. But when encountering EC17-preloaded FR+ (but not FR-negative) tumor cell targets, E2-CAR-T cells underwent significant differentiation with increased co-expression of exhaustion markers. Based on increased frequencies of triple- and double-positive cells, a more exhausted phenotype appeared on co-cultured CAR-T cells in the order of KB > HOS-FRα^fLuc^ > THP1-FRβ > MDA-MD-231. While EC17 dose-dependent apoptosis (Annexin V+) was seen in all FR+ tumor cell lines on day 2, high FR-expressing KB cells again showed disproportionally low apoptosis (Figure 4D).

### EC17-Mediated CAR-T Cell Expansion and Tumor Uptake *in vivo*

**Figure 5:**
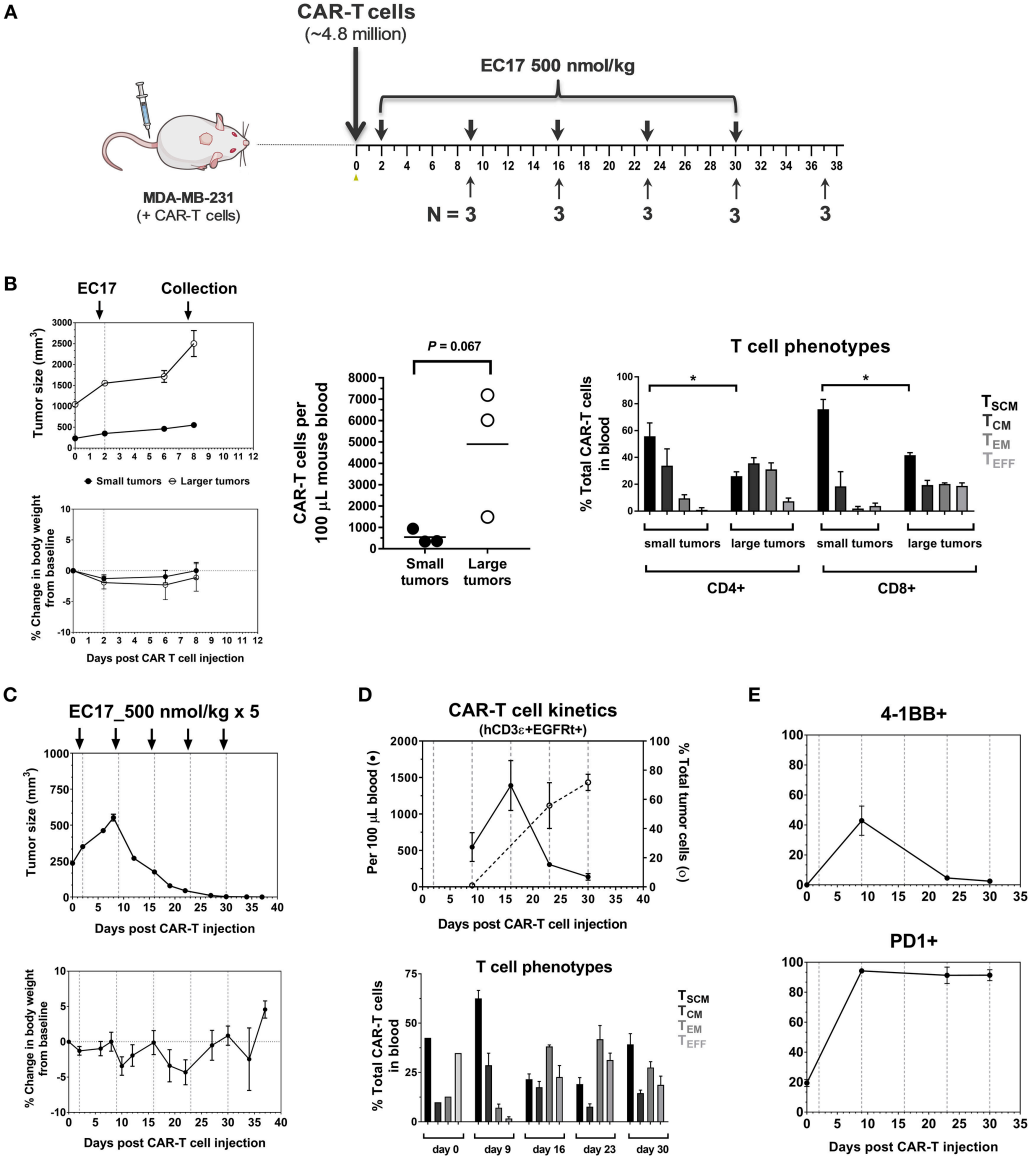
Pharmacokinetics and tumor uptake of CAR-T cells *in vivo*. **(A)** Schematic diagram of the experimental layout to show animal collection schedule in relation to CAR-T cell injection and weekly EC17 doses (500 nmol/kg) in MDA-MB-231 tumor-bearing mice. A total of 15 mice received ~4.8 million of EGFRt-sorted CAR-T cells on day 0 (3 mice had large tumors to begin with). **(B)** Left panel showing measurements of tumor volume and change in body weight; Middle plot showing CAR-T cell expansion in the blood 7 days after a single dose of EC17; Right bar graph showing differentiation profiles of circulating CD4/CD8 CAR-T cell subsets in mice with small and larger tumors. **(C)** Measurements of change in tumor volume and body weight. **(D)** Top plot showing CAR-T cell kinetics in the blood (solid line) vs. that of tumor (dotted line). Bottom bar graph showing changes in CAR-T cell phenotypes in the blood. **(E)** Kinetic changes in the surface expression of activation markers (4-1BB, PD1) on tumor-infiltrating CAR-T cells. *p < 0.05.

The dynamic interplay between EC17 and CAR-T cells *in vivo* was further interrogated using MDA-MB-231 tumor model, which was previously found to be among the highest FR expressing tumors at 103 ± 15 pmol/mg membrane protein using a ^3^H-FA-based radioligand binding assay (41). In our study, we evaluated the kinetics of CAR-T cell expansion in tumor-bearing mice that received ~4.8 million of EGFRt-sorted E2-CAR-T cells (Figure 5A). Seven days following a single EC17 dose (i.e., study day 9), human CD3ε+ EGFRt+ CAR-T cells were detected in the blood of mice, with elevated numbers circulating in mice bearing larger tumors (Figure 5B). In addition to trending toward a higher expansion *in vivo*, these blood borne CAR-T cells also exhibited a more differentiated profile (Figure 5B, 2504 ± 441 mm^3^ vs. 422 ± 16 mm^3^) in mice possessing larger tumors. Mice with the smaller sized tumors continued on study to receive a total of 5 weekly EC17 doses at 500 nmol/kg. As shown in Figure 5C, the MDA-MB-231 tumors started to respond after the second EC17 dose. Mice did experience mild body weight loss after EC17 administration, especially after the third dose. As circulating CAR-T cells peaked around day 15 and persisted for up to 30 days (last analysis by flow cytometry), we observed a steady increase in tumor-infiltrating CAR-T cells (Figure 5D, top plot, dashed line). In addition, CAR-T cells underwent dynamic phenotypic changes in the blood from a predominantly T_SCM_ phenotype on day 9, to more differentiated profiles on later days with continued EC17 treatment (Figure 5D, bottom bar graph). Accordingly, those tumor-infiltrating CAR-T cells were activated and expressed early (CD137/4-1BB) as well as late (PD1) T cell activation markers (Figure 5E). CD137/4-1BB expression peaked around day 9 while PD1 expression remained high from day 9 and forward. Thus, EC17 CAM dosing drives CAR-T cell activation, expansion and tumor uptake *in vivo* resulting a robust antitumor immunity.

### EC17 Dose Finding Safety Study in Tumor and Tumor-Free Mice

**Figure 6:**
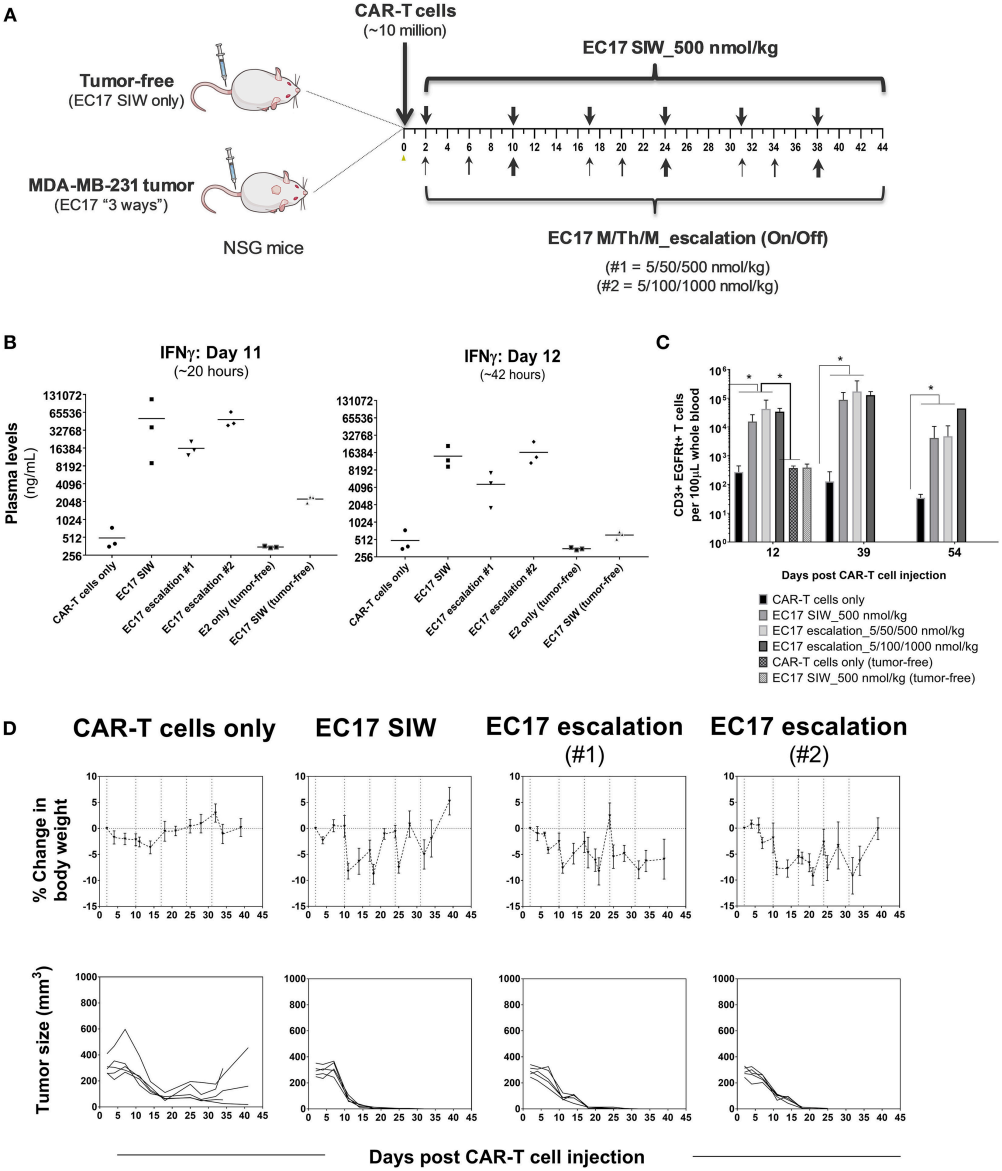
EC17 dose finding and CRS assessment in tumor vs. tumor-free mice. **(A)** Schematic diagram to show dose scheduling of CAR-T cells (~10 million “clinical facsimile”) plus EC17 dosed 3 different ways in NSG mice without or with pre-established MDA-MB-231 xenografts. Tumor-free mice received EC17 SIW 500 nmol/kg (2 total doses on days 2 and 10). Tumor-bearing mice received EC17 as follows: EC17 SIW 500 nmol/kg (5 total doses on days 2, 10, 17, 24, and 31), EC17 M/Th/M_Escalation-1 (repeats of 5/50/500 nmol/kg on Monday/Thursday/Monday followed by 1 week break, i.e., on days 2, 6, 10, 17, 20, 24, and 31), or EC17 M/Th/M_Escalation-2 (repeats of 5/100/1,000 nmol/kg on Monday/Thursday/Monday followed by 1 week break, i.e., days 2, 6, 10, 17, 20, 24, and 31). **(B)** Systemic levels of human IFNγ on a log2 scale detected in mouse plasma on days 11 and 12 after CAR-T cell injection in tumor-bearing vs. tumor-free mice (i.e., ~20 and 42 h after the previous EC17 dose in all treated cohorts). **(C)** Circulating CAR-T cells in mouse blood identified as human CD3ε+ EGFRt+ events by flow cytometry and enumerated per 100 μL of whole blood. **(D)** Measurements of change in body weight and tumor volume in tumor-bearing mice that received CAR-T cells only or CAR-T cells plus EC17 dosed 3 different ways (dashed lines indicated each EC17 dose). *n* = 5 mice per group. All data represent mean ± s.e.m. *p < 0.05 by one-way ANOVA test.

The initial EC17 dose finding studies were conducted in MDA-MB-231 tumor bearing mice as shown by the schematic diagram of the experimental layout (Figure 6A). NSG mice without or with MDA-MB-231 tumors (~211 ± 65 mm^3^) were engrafted on day 0 with ~10 million of a “clinical facsimile” batch of E2-CAR-T cells (~39% EGFRt+, 51:49 CD4/CD8). This batch of CAR-T cells consisted of mostly T_SCM_ and T_CM_ phenotypes (see Figure 1D). Two days after the CAR-T cell injection, one cohort of tumor-bearing mice were left untreated while three cohorts were treated with different regimens of EC17, including single injection per week (SIW) at 500 nmol/kg, or as escalating EC17 dose levels of 5/50/500 (Escalation-1) or 5/100/1000 nmol/kg (Escalation-2) given on a Monday/Thursday/Monday schedule with 1-week drug-free intervals. For comparison, two tumor-free cohorts were either untreated or treated with EC17 SIW at 500 nmol/kg.

Human T cell-derived IFNγ levels in mouse blood were measured in all cohorts using satellite animals on days 11 and 12 (~20 and 42 h after the previous EC17 dose). Compared to tumor-bearing mice that received CAR-T cells only, all EC17 treated tumor cohorts had ~30x (day 11) and ~10x (day 12) higher IFNγ production in mouse plasma, and the levels of this cytokine decreased naturally from 20 to 42 h later (Figure 6B). In accordance with IFNγ release, EC17 also triggered CAR-T cell expansion identified as human CD3ε+ EGFRt+ events in mouse blood, and cells persisted up to 54 days in tumor-bearing animals (last measurement) (Figure 6C). In tumor-free cohorts, no CAR-T cell expansion was detected by flow cytometry, but low levels of IFNγ were detected on days 11 and 12 in animals that received the same number of CAR-T cells plus EC17 (Figures 6B,C). Moreover, no CRS symptoms (grade 0 out of a 0–5 scale) or body weight loss was observed in tumor-free mice that received 2 weekly doses of EC17 at 500 nmol/kg.

Moderate-to-severe CRS symptoms (grades 2-3) and significant body weight losses (Figure 6D) were observed in tumor-bearing cohorts with continued EC17 dosing independent of the regimen. While EC17 SIW at 500 nmol/kg caused the earliest onset of CRS and body weight loss, the aggressive EC17 Escalation-2 regimen (up to 1,000 nmol/kg) caused persistent body weight loss with animal recovery occurring after EC17 dose cessation (Figure 6D, top row). Notably, symptoms of graft-vs.-host disease (GVHD) included red itching skin and hairlessness became obvious at ~1 month after CAR-T cell engraftment. Although animals receiving only CAR-T cells showed signs of nonspecific CAR-T cell/tumor alloreactivity, only EC17-treated cohorts produced 100% cures (Figure 6D, bottom row). Therefore, EC17 administration in the presence of FR+ tumors was the key to drive (i) CAR-T cell activation, (ii) cytokine production, and (iii) *in vivo* CAR-T cell expansion and persistence. Under specific conditions, however, severe CRS (grade ≥ 3) was triggered by a high CAR-T cell dose in combination with EC17 doses equal or >500 nmol/kg (from herein referred to as 100% full dose).

### Effect of High Dietary Folate on CRS and Antitumor Activity

**Figure 7:**
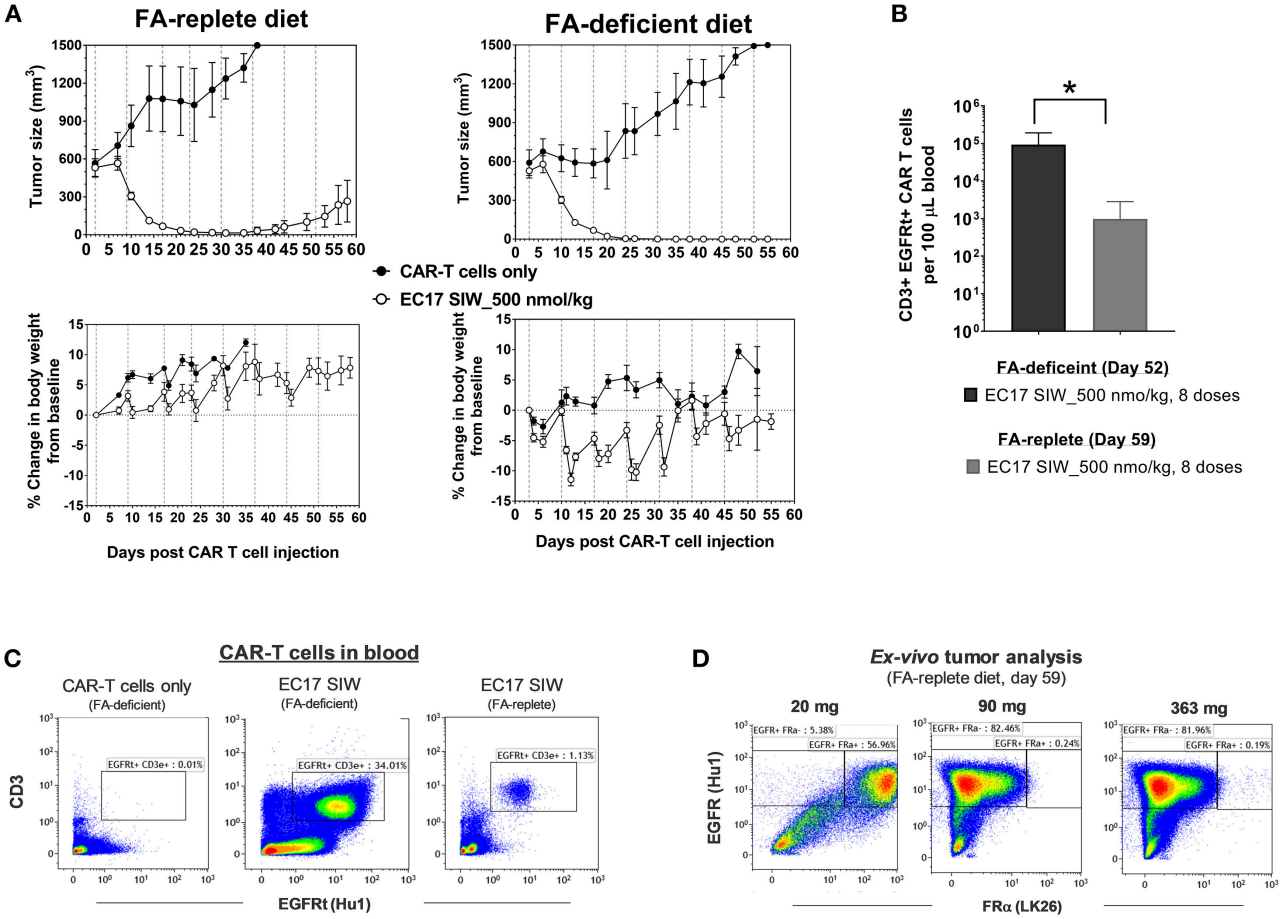
Dietary folate effect on antitumor activity and CRS toxicity. **(A)** Measurements of change in tumor volume and body weight of NSG mice xenografted with large MDA-MB-231 tumors and treated with CAR-T cells (~10 million “clinical facsimile”) plus EC17 SIW at 500 nmol/kg while being maintained on FA-replete or FA-deficient diet throughout the study (*n* = 5). **(B)** Bar graph to show circulating CAR-T cells (human CD3ε+ EGFRt+) measured at the end of study on day 52 (FA-deficient) and day 59 (FA-replete), respectively. **(C)** Representative flow cytometric dot plots to show an absence of circulating CAR-T cells in FA-deficient mice treated with CAR-T cells only (no EC17) and elevated numbers of circulating CAR-T cells in mice treated with CAR-T cells plus EC17 on FA-deficient diet compared to mice treated identically but on FA-replete diet. **(D)** Loss of FR expression was detected in 2 out of 3 MDA-MB-231 tumors collected on day 59 from FA-replete animals and analyzed by flow cytometry (later confirmed by a quantitative radioligand binding assay, data not shown). *p < 0.05.

Previously, it was shown that administration of FA or a FA ligand helped reduce the severity of CRS in MDA-MB-231 tumor-bearing mice (27, 33). To test the long-term effect of dietary folate, mice were maintained on defined diets for ~73 days and used when their tumors reached ~549 ± 184 mm^3^ (280–918 mm^3^) in FA-replete mice, and ~559 ± 165 mm^3^ (356–961 mm^3^) in FA-deficient mice (Figure 7). All mice received ~10 million of the same “clinical facsimile” of E2-CAR-T cells on day 0, and EC17-treated cohorts received 8 weekly doses of 500 nmol/kg starting on day 2 (FA-replete) or day 3 (FA-deficient). As seen previously, the first full dose of EC17 was generally safe and did not cause any CRS or body weight loss in mice on either diet. Actually, FA-replete animals did not show symptoms of CRS throughout EC17 treatment, whereas FA-deficient animals experienced grades 2–3 CRS and body weight loss (up to ~11.4%) with each of the subsequent EC17 doses (Figure 7A, bottom panels). In the same duration of EC17 treatment, a much higher level of CD3ε+ EGFRt+ CAR-T cells was found in mice on FA-deficient diet (1.6–20.4 × 10^4^ per 100 μL blood on day 52) when compared to mice on FA-replete diet (0.027–4.3 × 10^3^ per 100 μL blood on day 59) (Figures 7B,C). Importantly, there was no CAR-T cell expansion detected in FA-deficient animals that did not receive EC17 treatment (Figure 7C).

Since we started MDA-MB-231 tumors at larger sizes than the previous study (see Figure 6), tumor regression due to alloreactivity at this 10-million “clinical facsimile” CAR-T cell dose was not apparent (Figure 7A, top rows). Control tumors (no EC17) in FA-replete mice appeared to have faster growth kinetics compared to control tumors in FA-deficient mice; but overall, there were 2/5 cures, 2/5 complete responses, and 1/5 partial response in FA-replete mice that did receive EC17 (Figure 7A, top left figure). A more robust *in vivo* T cell expansion was seen in mice on FA-deficient diet and resulted in 5/5 cures, although these animals' health deteriorated with each CRS episode (Figure 7A, top right figure).

Using anti-human EGFR (clone Hu1) to identify MDA-MB-231 cancer cells within mouse tumor masses after an enzymatic digestion, we analyzed FRα protein expression (clone LK26) in three tumors recovered from mice on FA-replete diet that experienced a relapse despite continuing EC17 treatment. By flow cytometric detection, EGFR+ cancer cells isolated from the smallest tumor (21 mg) expressed FRα, while EGFR+ cancer cells isolated from the two larger tumors (90 and 363 mg) had completely lost their FRα expression (Figure 7D). Using the quantitative radioligand binding assay with ^3^H-FA, we further confirmed the loss of functional FR levels on tissue homogenates obtained from these tumors (~2.1 pmol/mg binding potential vs. ~103 pmol/mg in tumors from untreated animals). Therefore, FA-deficiency may lead to enhanced activity and CRS toxicity; while non-physiological FA intake prevents CRS and continuous consumption may lead to reduced activity (see Discussion).

### EC17 CAM Dosing Control in Anti-leukemic Activity

**Figure 8:**
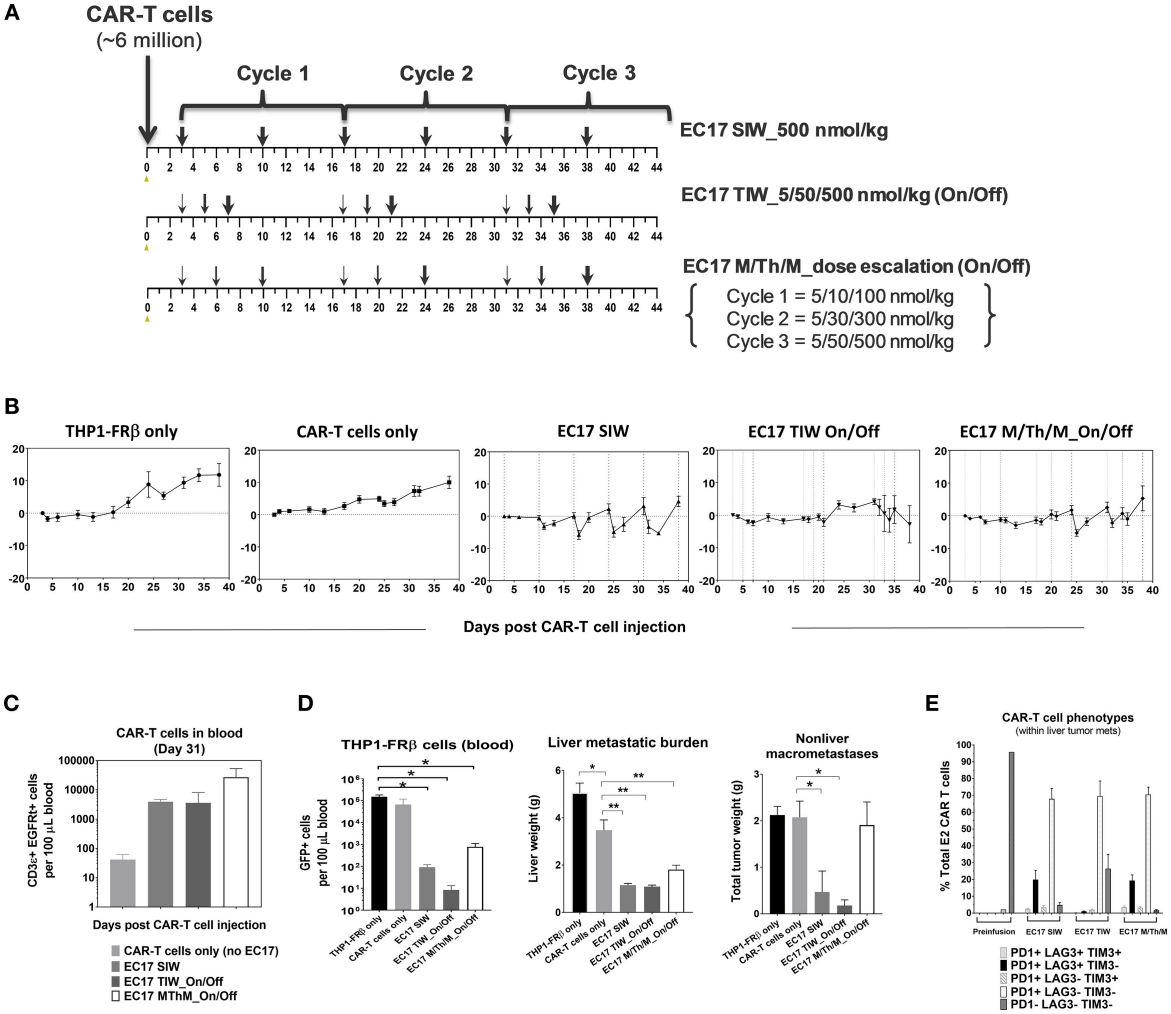
EC17 dose escalation in safety and anti-leukemic activity *in-vivo*. **(A)** Schematic diagrams to show dose scheduling of EC17 plus unsorted EGFRt CAR-T cells (~6 million, day 0) in NSG mice with 1 day-old intravenous THP1-FRβ xenografts. Starting 3 days after CAR-T cell injection, EC17 was dosed in 3 different ways: EC17 SIW 500 nmol/kg (5 total doses on days 3, 10, 17, 24, 31, and 38), EC17 TIW 5/50/500 nmol/kg (3 repeats of 5/50/500 nmol/kg on Monday/ Wednesday/Friday followed by a 9 day break, i.e., on days 3/5/7, 17/19/21, and 31/33/35), or EC17 M/Th/M_dose escalation on Monday/Thursday/Monday (3 escalation cycles at 5/10/100 nmol/kg in Cycle 1, 5/30/300 nmol/kg in Cycle 2, and 5/50/500 nmol/kg in Cycle 3, i.e., on days 3/6/10, 17/20/24, and 31/34/38). **(B)** Measurements of change in body weight (*n* = 5). **(C)** Circulating CAR-T cells as human CD3ε+ EGFRt+ events found per 100 μL of mouse whole blood on a logarithmic scale on day 31. **(D)** Left bar graph: circulating tumor cells (GFP+) per 100 μL of whole blood in all cohorts measured at the end of study (day 39); Middle bar graph: THP1-FRβ infiltrated liver weights representing liver metastatic burden; Right bar graph: total tumor weights of all non-liver macrometastases. **(E)** Flow cytometric analysis of T-cell exhaustion markers, PD1, LAG3, TIM3, on preinfusion CAR-T cell product (triple-negative) and tumor-infitrating CAR-T cells isolated from liver metastases. A cardinal feature of fully exhausted T cells is co-expression of multiple inhibitory receptor markers (i.e., triple-positive) (39). *p < 0.05; **p < 0.01.

Intravenously implanted GFP-expressing THP1-FRβ tumor cells developed disseminated diseases in NSG mice with tumor cells in the circulation and liver/non-liver metastases throughout the body. We found that THP1-FRβ tumor cells could also localize to the mouse ovary which appeared inflamed during the early stage of tumor progression. Therefore, total tumor burden in each animal in study cohorts was assessed by quantitating circulating GFP+ tumor cells in the blood, liver weights, and all-inclusive non-liver macrometastases visible to the naked eye. Although THP1-FRβ expressed a low level of FR *in vitro*, THP1-FRβ tumor metastases were found to express a higher than expected functional FRs level at ~8.9 ± 2.8 pmol/mg membrane protein. Thus, THP1-FRβ tumor-bearing mice were engrafted with a research batch of EGFRt-unsorted E2-CAR-T cells (~23% EGFRt+, 1:1 CD4:CD8) at ~6 million/animal and then treated with 3 different EC17 dosing regimens (Figure 8). Starting 3 days after CAR-T cell injection, EC17 dosing regimens began as SIW at 500 nmol/kg continuously, thrice a week (TIW) at 5/50/500 nmol/kg on Monday/Wednesday/Friday followed by a 9 day break, or by an accelerated dose escalation regimen at 5/10/100 nmol/kg in Cycle 1, 5/30/300 nmol/kg in Cycle 2, and 5/50/500 nmol/kg in Cycle 3, on Monday/Thursday/Monday (M/Th/M) followed by a 6 day break (Figure 8A). While some body weight loss and grade 1–2 CRS in Cycles 2 and 3 were observed in animals received EC17 SIW treatment, animals that received EC17 TIW had the least body weight loss with grade 0–1 CRS only (Figure 8B). Amongst the animals that received the 3 cycles of EC17 M/Th/M dose escalation, grade 0–1 CRS and very mild body weight loss were observed in Cycle 2 after the last dose of EC17 of 300 nmol/kg. Using satellite animals on day 31, we enumerated CAR-T cells in the blood and demonstrated EC17-dependent CAR-T cell expansion and persistence in all treated cohorts (Figure 8C). Compared to control animals that received tumor cells only or tumor cells plus CAR-T cells without EC17, EC17 dosed with any of the 3 “intra-patient” escalation formats effectively reduced circulating THP1-FRβ tumor cells in the blood and showed similar activities against liver tumor metastases (Figure 8D, left and middle bar graphs). Only minor allogeneic reactivity was seen against THP1-FRβ liver metastases in mice that received CAR-T cells only. While EC17 SIW and TIW at 10-fold dose escalation (on/off) successfully controlled non-liver macrometastases, EC17 M/Th/M dose escalation at a slow pace per cycle (Figure 8A) failed to control the non-liver macrometastases (Figure 8D, far right panel). At the end of study (i.e., 39 days post CAR-T cell injection), CAR-T cells isolated from liver THP1-FRβ tumor metastases of EC17 TIW-treated animals appeared to have the least expression of double- and triple-positive T cell inhibitory receptors, PD1, LAG3, and TIM3 (Figure 8E). Overall, no severe CRS (i.e., grades ≥ 3) was observed in any EC17 treated cohorts. Nevertheless, the more aggressive EC17 TIW dose escalation (on/off) trended as the best regimen for reducing overall THP1-FRβ tumor burden in these mice.

### A Dose Escalation Trial Against an Aggressive Osteosarcoma Model

**Figure 9:**
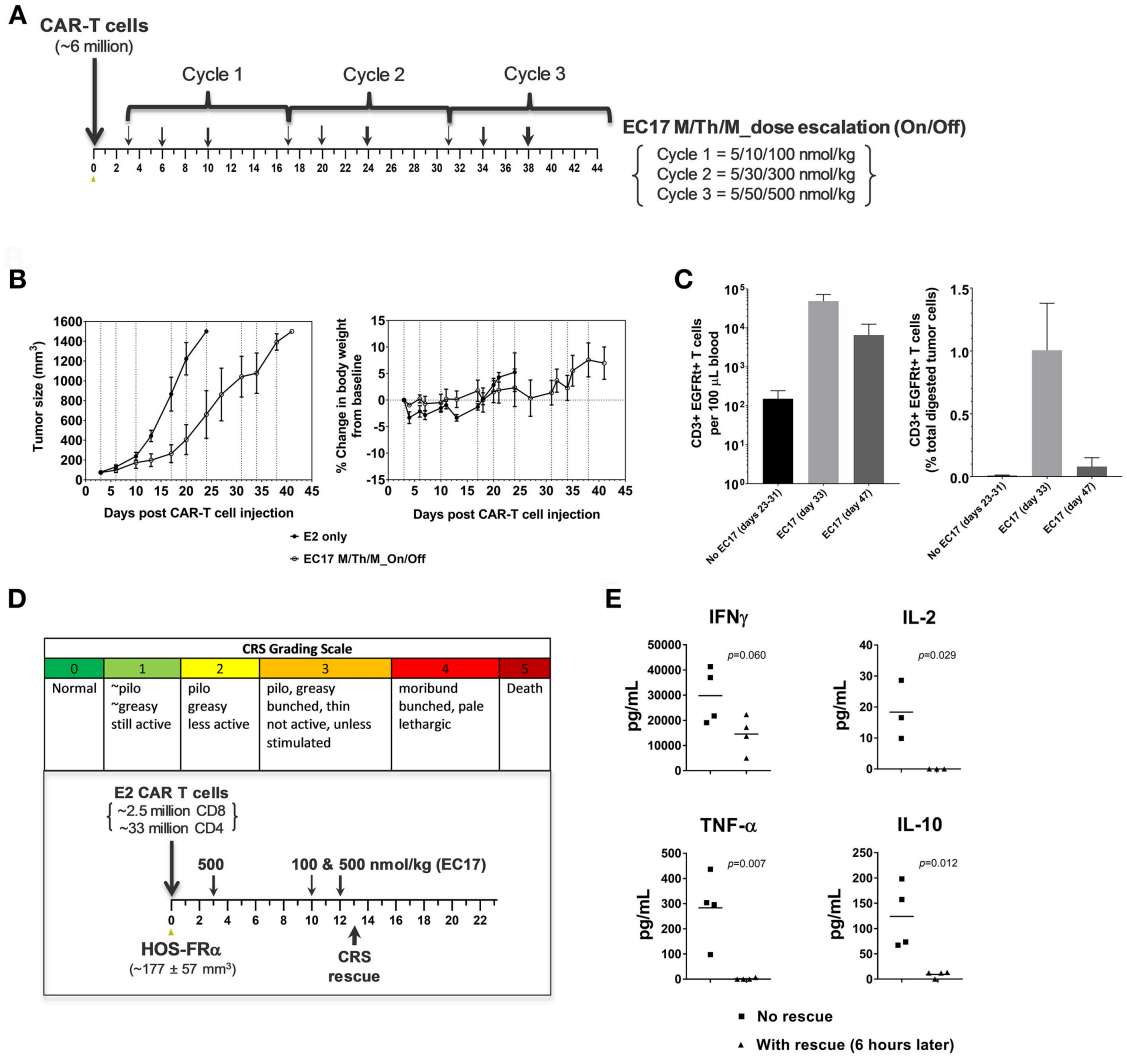
Antitumor activity and CRS rescue in an aggressive model of pediatric osteosarcoma. **(A)** Schematic diagram to show dose scheduling of CAR-T cells (~6 million, day 0) plus EC17 in NSG mice with 3 day-old subcutaneous HOS-FRα xenografts (*n* = 5). Starting 3 days after CAR-T cell injection (6 days after tumor implant), 3 cycles of EC17 M/Th/M “intra-patient” dose escalation on Monday/Thursday/Monday at 5/10/100 nmol/kg in Cycle 1, 5/30/300 nmol/kg in Cycle 2, and 5/50/500 nmol/kg in Cycle 3, i.e., on days 3/6/10, 17/20/24, and 31/34/38. **(B)** Measurements of tumor volumes and change in body weights. Due to tumor progression, five mice received CAR-T cells only (no EC17) were euthanized on days 23–31, and five EC17 treated mice were euthanized on days 33 (2 mice) and 47 (3 mice), respectively. **(C)** Flow cytometric analysis of CAR-T cells (human CD3ε+EGFRt+) per 100 μL of whole blood plotted on a logarithmic scale (left) and tumor-infiltrating CAR-T cells at the time of euthanasia (right). **(D,E)** CRS grading scale and experimental conditions applied to sodium fluorescein rescue in HOS-FRα tumor-bearing mice. Shown are changes in T cell-derived cytokines with and without rescue. *p*-values were calculated by Student's *t*-test.

For our intended purpose, HOS-FRα is a low FR-expressing but most aggressive tumor model with a functional FR level of ~5.82 ± 1.45 pmol/mg protein. In parallel to the THP1-FRβ study described above, two cohorts of mice with 3 day-old HOS-FRα tumors were given the same E2-CAR-T cells at the same dose (~6 million). One cohort was treated with the same accelerated EC17 dose escalation regimen at 5/10/100 nmol/kg in Cycle 1, 5/30/300 nmol/kg in Cycle 2, and 5/50/500 nmol/kg in Cycle 3, on Monday/Thursday/Monday schedule followed by a 6 day break (Figure 9A). As HOS-FRα tumors grew aggressively without EC17 treatment, accelerating EC17 dose escalation at this CAR-T cell dose was safe (no CRS or body weight loss) and resulted in a significant delay in tumor growth within the first two cycles of treatment (Figure 9B). Upon protocol-mandated euthanasia due only to tumor size (≥1,500 mm^3^), flow cytometric analyses performed on whole blood showed an EC17-dependent CAR-T cell expansion up to day 47 but higher on day 33 (Figure 9C, left bar graph). *Ex vivo* tumor analysis on day 33 indicated a low but significant intratumoral CD3ε+ EGFRt+ CAR-T cell population in EC17-treated animals which amounted to ~1% total viable digested tumor-derived cells (Figure 9C, right bar graph). As large HOS-FRα tumors stopped responding to treatment in Cycle 3 (Figure 9B), intratumoral CAR-T cells also diminished on day 47 (Figure 9C). Notably, HOS-FRα tumors analyzed by ^3^H-FA radioligand assay upon disease progression showed similar FRα levels with and without EC17 treatment. Thus, it appeared that HOS-FRα tumors in NSG mice quickly outgrew the tumor infiltrating capability of CAR-T cells and may have decreased cytolytic activity due to T cell exhaustion as suggested by *in vitro* co-culture studies (Figure 4B).

To scrutinize our CRS rescue capabilities with sodium fluorescein, we developed a CRS scoring system and was able to capture the onset of severe CRS (grades 3/4) following EC17 administration (Figure 9D). As shown by a challenging case of rapid CRS onset in HOS-FRα tumor-bearing mice due to a very high dose of 35 million total CAR-T cells (93:7 CD4/CD8), intravenous sodium fluorescein at a human equivalent dose (~95 mg/kg in mice) acted very quickly to reduce CAR-T cell derived human cytokines (Figure 9E).

## Discussion

In this study, we employed humanized FITC-E2 CAR and CAR-modified T cells (1:1 CD4/CD8) that were rationally designed and produced to meet clinical requirements (Figure 1D). Based on work by Ben Towne Center for Childhood Cancer Research and Fred Hutchinson Cancer Research Center, CAR-T cell product at a defined 1:1 CD4/CD8 ratio and limited effector differentiation offered several advantages including: (i) better correlation between cell dose and CAR-T cell expansion/ persistence, (ii) better prediction and mitigation of toxicity; and (iii) potentially superior therapeutic efficacy in patients (42, 43). Using CAR-T cells provided by the Jensen laboratory, we performed *in vitro* and *in vivo* studies to elucidate pharmacological and pharmacokinetic properties of EC17 CAM action with a special focus on pediatric cancer models. At low pico-molar EC50s in co-culture systems, EC17 triggers a potent Th1 cytokine production and FR-dependent target cell lysis by E2-CAR-T cells (Figures 2, 3). For the most part, a semi-log positive correlation was established between FR expression levels and EC17 CAM-elicited Th1 responses from CAR-T cells. However, total functional FR levels expressed by tumor cells and tumor cells' intrinsic sensitivity/resistance to T cell therapy likely dictate a maximal possible response and tumor rejection rate (Figure 3). For sensitive but low FR-expressing cell targets such as OV90, it required more effector cells (e.g., E/T 1:1) and longer response time (48 h) to show any significant killing in the presence of EC17. Although a FR threshold has yet to be determined *in vitro*, low EC50s and receptor occupancy of EC17 CAM on FR+ cancer cells suggest that not all FRs need to be occupied to trigger a potent CAR-T cell response. When encountering different FR+ tumor targets, CAR-T cells exhibited different activation and exhaustion profiles and became more exhausted more quickly with certain tumor types (e.g., KB) (Figure 4). This goes beyond functional FR levels and helps explain why MDA-MB-231 (~2.1 × 10^6^ FA molecules/cell) was the most sensitive of all cancer cell lines tested. KB (~4.8 × 10^6^ FA molecules/cell) was the least sensitive, slowest to respond and required a higher E/T ratio to commence killing (Figure 3). Interestingly, our FR+ HOS-FRα^fLuc^ cell line expresses only ~240,000 FR molecules/cell which was more than enough to trigger a potent CAR-T cell response in short-term cultures (Figures 2, 3). However, like HOS-143b-derived cell lines, HOS-FRα^fLuc^ grew aggressively in culture (~1 day doubling time), a quick drop in E/T ratio was likely the cause for a more exhausted CAR-T cell profile (Figure 4C). Further studies are needed to determine what co-inhibitory signals on FR+ cancer types, such as KB, that lend its resistance to CAR-T cell mediated apoptosis (Figure 4E).

It has been reported how fast tumor cells are killed by CAR-T cells *in vivo* contributes to long-term T cell proliferation and tumor rejection rate (44). In our bispecific approach, EC17 CAM provides the tumor antigen (i.e., FITC) and how it is given controls the rate of CAR-T cell expansion in tumor-bearing hosts. In the MDA-MB-231 tumor model, we found that weekly EC17 doses at 500 nmol/kg starting 2 days after CAR-T cell infusion induced a rapid CAR-T cell expansion with a trending positive correlation with tumor burden as elevated CAR T cell numbers were observed in the blood from mice with larger tumors (Figure 5B). The number of CAR-T cells in the circulation then peaked around 15 days and persisted for at least 30 days (Figure 5D). In accordance with a surge of CAR-T cells in the circulation, MDA-MB-231 tumors started to shrink after the second weekly EC17 dose, and they eventually became fully infiltrated with CAR-T cells (Figures 5C,D). Flow cytometric analysis of tumor-infiltrating CAR-T cells indicated that these CAR-T cells were activated with elevated expression of CD137/4-BB and PD1 (Figure 5E). Similar studies are being planned to use high FR-expressing but more resistant tumor models, such as KB.

Commercial rodent diets contain high amount of folates, including the synthetic fully oxidized form, FA (45). An approximate 40-fold difference in serum folate levels was reported between mice on FA-replete diet (~800 nM) and an average human adult (~20 nM) (46). Because excess FA in mouse blood serves as a competitor for EC17 and can occupy tumor FRs, we routinely placed NSG mice on FA-deficient diet to lower their serum folate levels (~15 nM) before initiation of EC17 treatment. In MDA-MB-231 tumor-bearing mice, a high level of functional FRs (~103 ± 15 pmol/mg protein) was maintained on both diets, and tumors as large as ~600 mm^3^ were found sensitive to CAR-T cell therapy with weekly EC17 dosing (Figure 7A). Although we did not closely follow CAR-T cell expansion and persistence in real-time, mice with high dietary folate appeared to have lower levels of CAR-T cells at the end of study corresponding to a decreased cure rate (Figures 7B,C). This is likely because high levels of FA in the circulation interfered with EC17 binding and perpetuated a constant disruption of target engagement by CAR-T cells. In mice on FA-replete diet, the loss of FRα expression on two MDA-MB-231 tumors (despite continuing EC17 treatment) suggested a potential mechanism for cancer cells to evade immune attack (4). Therefore, serum folate status in patients should be monitored and non-physiological intake of FA should be discouraged in clinic settings to avoid competition against the EC17 CAM.

Depending on cancer subtypes and histology, FR+ malignant tissues do not necessarily maintain FR function following serial passage in animals and/or tissue culture. Unfortunately to represent pediatric malignancies, we are limited by the availability of FRα/β+ tumor models in-house and from commercial sources. Using THP1-FRβ and HOS-FRα xenografts to represent pediatric AML and osteosarcoma, respectively, we compared them against MDA-MB-231 in sensitivity to EC17 CAM-controlled CAR-T cell therapy. While intravenous THP1-FRβ formed both liquid and solid tumors in NSG mice, subcutaneous HOS-FRα tumors grew aggressively reaching ~1,200 mm^3^ in ~17 days (35). Unlike MDA-MB-231 tumors which were usually 100% curable regardless of how EC17 was given, no cures were observed in both pediatric models (Figures 8, 9). Our co-culture data suggested that THP1-FRβ cells were naturally sensitive to T cell-mediated killing; however, once established outside of the circulation, extramedullary THP1-FRβ metastases behaved more like solid tumors and became more difficult to treat (Figure 8D). Compared to a slow EC17 M/Th/M dose escalation (i.e., not reaching the full dose until day 38), weekly EC17 dosing at its full dose (500 nmol/kg) or EC17 TIW at 10-fold dose escalation (5/50/500 nmol/kg, on and off with a 9 day resting period) was significantly more effective at reducing overall THP1-FRβ tumor burden (Figure 8D). Tumor-infiltrating CAR-T cells (liver metastases) from EC17 TIW treated cohorts also appeared to have the least expression of double- or triple-positive surface inhibitory receptors, PD1, LAG3, and TIM3 (Figure 8E). This suggests that tumor-induced CAR-T cell exhaustion may be prevented by controlling antigen exposure kinetics with a EC17 CAM dosing strategy. Although tumor microenvironment in NSG mice is very different from that of a patient, lessons from our xenograft studies suggest that solid tumors may possess mechanisms of resistance other than low target expression. For example, differences in blood supply and tumor microenvironment may explain why circulating THP1-FRβ cells and liver metastases were more sensitive to treatment than non-liver metastases in the same animal. We derived HOS-FRα from one of the most aggressive osteosarcoma cell line HOS-143b (35). Its aggressive growth rate in severely immunodeficient NSG mice may not reflect that of a majority of osteosarcoma patients with metastatic diseases. Taken together, our *in vitro* and *in vivo* findings seemed to be in good agreement suggesting a tumor elimination kinetics in an order of MDA-MB-231 > THP1-FRβ > HOS-FRα. While a high FR expression is obviously desirable, improving CAR-T cell trafficking into tumor microenvironment would be important to offset a low E/T ratio and T cell exhaustion.

Regarding safety and CRS toxicity, the first full-dose of EC17 (500 nmol/kg) given 2–3.5 days after CAR-T cell administration was safe with no CRS symptoms or body weight loss in tumor-bearing mice. In the MDA-MB-231 tumor model, E2-CAR-T cells were highly active and expanded dramatically with EC17 administration. However, severe CRS occurred in FA-deficient animals that received a high CAR-T cell dose (~10 million) plus EC17 at its full dose during the second and third weeks of CAR-T cell expansion. Although these CAR-T cells showed nonspecific T cell/tumor alloreactivity, EC17-dependent antitumor activity was clearly demonstrated against large MDA-MB-231 tumors (Figures 7, 8). Notably, equivalent numbers of E2-CAR-T cells did not cause CRS in tumor-bearing mice without EC17 treatment or in tumor-free mice with EC17 treatment. Nevertheless, too high of a CAR-T cell dose should be avoided as it increased the incidence and severity of CRS toxicity upon continuing EC17 treatment (Figures 7, 8). Once we dropped the CAR-T cell dose to ~5–6 million/animal for THP1-FRβ and HOS-FRα studies, EC17 CAM dosing was safer and its tumor uptake more easily modulated to minimize the risk of severe CRS by means of intermittent “on-and-off” and intra-host dose titration (Figures 8, 9). As high dietary folic acid diminished the risk of CRS while maintaining significant activity against MDA-MB-231, we can potentially use lower affinity folates (e.g., leucovorin) for CRS rescue (33). Previously with a different CAR construct, we discovered that CAR-T cells moved out of circulation following each EC17 dose while sodium fluorescein dose-dependently returned 4-1BB+ CAR-T cells to the blood (27). Future studies are planned to closely monitor CAR-T cell activation kinetics in the blood vs. that of tumor and to investigate the opposite effect of EC17 and sodium fluorescein in target engagement/disruption by immunohistochemistry. It is worth mentioning that FR expression levels on normal tissues are generally low and restricted to luminal or apical surfaces of a few organs where CAR-T cells would not be accessible under normal physiological conditions (47).

Recently, medulloblastoma was found to have a high frequency of FRα protein expression (48), and depending on the subtypes some may have a complete breakdown of blood-brain barrier (49, 50). A second-generation HER2-BBζ-CAR has shown excellent pre-clinical activities in orthotopic mouse models (51) and is currently under clinical investigation (ClinicalTrials.gov identifier: NCT03500991). A FR+ subtype of medulloblastoma could be another potential childhood indication that a CAM controlled FRα-specific CAR-T cell therapy may find its potential utility. Moreover, immunosuppressive TAMs can impede CD8 T cells from reaching tumor cells and has limited the efficacy of anti-PD-1 treatment (52). In preclinical models, anti-CD123 CAR-T cells recognize and kill TAMs in Hodgkin lymphoma and enhanced antitumor activity (53). Since a subset of TAMs also express functional FRβ, EC17 CAM may have dual utilities in certain tumor types or metastatic settings by engaging CAR-T cell killing of both malignant cells and unfavorable FRβ+ TAMs.

## Conclusion

Current standard practice in treating pediatric solid tumors replies on surgery and multiple rounds of “heavy-duty” chemotherapy to control the disease. Once the disease relapses, treatment options are very limited with unsatisfactory results. As T cells are of better quality in kids and young adults, the success of CD19-directed CAR-T cell therapies against childhood B-cell malignancies underscores the potential of CAR-T cell therapy against some forms of pediatric solid tumors. Our CAM-controlled CAR-T cell therapy platform offers multiple levels of antigen control in regards to CAR-T cell function/exhaustion, safety and antitumor activity. In preclinical models, EC17 exhibits a potent bispecific activity, but EC17-orchestrated insult to FR+ tumor cells is governed by the magnitude of FR expression and how fast and how efficient tumor cells are killed by CAR-T cells on site. As autologous CAR-T cell therapies are highly individualized, correlative studies may be used to guide EC17 CAM administration in the clinic. Further, combinations and loco-regional delivery are important in dealing with tumor cells' natural sensitivity, tumor microenvironment, and other common factors that can limit CAR-T cell efficacy in solid tumors.

## Data Availability

All datasets generated for this study are included in the manuscript and/or the supplementary files.

## Ethics Statement

The animal protocol was approved by, and the studies within carried out in accordance with the recommendations of the Purdue Animal Care and Use Committee at Purdue University.

## Author Contributions

The MJ's team conceived, engineered, and selected the clinical CAR construct and provided CAR-modified T cells produced under proprietary conditions. The Endocyte team conducted chemical synthesis, conceived, designed, and conducted pharmacokinetic and pharmacodynamic studies *in vitro* and *in vivo*. YL wrote the paper. CL edited the paper. All authors reviewed the manuscript.

### Conflict of Interest Statement

All authors listed as employees of Endocyte Inc. (YL, HC, LW, MN, EW, NP, MV, L-CX, EZW, PK, PL, CL) were stock holders at the time this work was completed. The remaining authors declare that the research was conducted in the absence of any commercial or financial relationships that could be construed as a potential conflict of interest.
